# Paleoproteomic evidence reveals dairying supported prehistoric occupation of the highland Tibetan Plateau

**DOI:** 10.1126/sciadv.adf0345

**Published:** 2023-04-12

**Authors:** Li Tang, Shevan Wilkin, Kristine Korzow Richter, Madeleine Bleasdale, Ricardo Fernandes, Yuanhong He, Shuai Li, Michael Petraglia, Ashley Scott, Fallen K.Y. Teoh, Yan Tong, Tinlei Tsering, Yang Tsho, Lin Xi, Feng Yang, Haibing Yuan, Zujun Chen, Patrick Roberts, Wei He, Robert Spengler, Hongliang Lu, Shargan Wangdue, Nicole Boivin

**Affiliations:** ^1^ Department of Archaeology, Max Planck Institute for the Science of Human History, Jena, Germany.; ^2^ Center for Archaeological Science, Sichuan University, Chengdu, China.; ^3^ Institute for Prehistoric and Protohistoric Archaeology, Kiel University, Kiel, Germany.; ^4^ Australian Research Centre for Human Evolution, Griffith University, Brisbane, Australia; ^5^ Institute for Evolutionary Medicine, University of Zürich, Zürich, Switzerland.; ^6^ Department of Anthropology, Harvard University, Cambridge, USA.; ^7^ Department of Archaeology, University of York, York, UK.; ^8^ Department of Archaeology, Max Planck Institute of Geoanthropology, Jena, Germany.; ^9^ Faculty of Arts, Masaryk University, Brno, Czech Republic.; ^10^ Climate Change and History Research Initiative, Princeton University, Princeton, NJ, USA.; ^11^ School of Archaeology and Museology, Sichuan University, Chengdu, China.; ^12^ Center for Tibetan Studies, Sichuan University, Chengdu, China.; ^13^ School of Social Science, University of Queensland, Brisbane, Australia.; ^14^ Human Origins Program, National Museum of Natural History, Smithsonian Institution, Washington, DC, USA.; ^15^ Department of Archaeogenetics, Max Planck Institute for Evolutionary Anthropology, Leipzig, Germany.; ^16^ Department of Life Sciences and Systems Biology, University of Turin, Turin, Italy.; ^17^ Tibetan Cultural Relics Conservation Institute, Lhasa, China.; ^18^ Shaanxi Academy of Archaeology, Xian, China.; ^19^ isoTROPIC Research Group, Max Planck Institute of Geoanthropology, Jena, Germany.; ^20^ Domestication and Anthropogenic Evolution Research Group, Max Planck Institute of Geoanthropology, Jena, Germany.; ^21^ Department of Anthropology, National Museum of Natural History, Smithsonian Institution, Washington, DC, USA.; ^22^ Griffith Sciences, Griffith University, Brisbane, Australia.

## Abstract

The extreme environments of the Tibetan Plateau offer considerable challenges to human survival, demanding novel adaptations. While the role of biological and agricultural adaptations in enabling early human colonization of the plateau has been widely discussed, the contribution of pastoralism is less well understood, especially the dairy pastoralism that has historically been central to Tibetan diets. Here, we analyze ancient proteins from the dental calculus (*n* = 40) of all human individuals with sufficient calculus preservation from the interior plateau. Our paleoproteomic results demonstrate that dairy pastoralism began on the highland plateau by ~3500 years ago. Patterns of milk protein recovery point to the importance of dairy for individuals who lived in agriculturally poor regions above 3700 m above sea level. Our study suggests that dairy was a critical cultural adaptation that supported expansion of early pastoralists into the region’s vast, non-arable highlands, opening the Tibetan Plateau up to widespread, permanent human occupation.

## Teaser

Dairy pastoralism began on the plateau by ~3500 years ago and supported population expansion into its vast, non-arable highlands.

## INTRODUCTION

The Tibetan Plateau, as the world’s highest and largest plateau, represents one of the most inhospitable global environments to which humans have adapted. Referred to as the “third pole,” the high mountain region has more snow and ice than anywhere on Earth except for the north and south poles (*1*). The plateau’s combination of cold temperatures, hyper-aridity, and unpredictable weather results in a scarcity of food resources in most of its highland regions while also demanding higher caloric intake to sustain human metabolic activities (*2*). In addition, the plateau’s low atmospheric pressure causes hypoxia, a shortness of oxygen in the body (*3*, *4*), that can be lethal to humans. Although positive natural selection at several genomic loci (e.g., *EPAS1*, *EGLN1*, and *MTHFR*) enabled early Tibetans to adapt biologically to living at high elevations (*5*–*8*), the challenge of acquiring sufficient food from the barren heights of the plateau also required cultural adaptations.

**Fig. 1.**
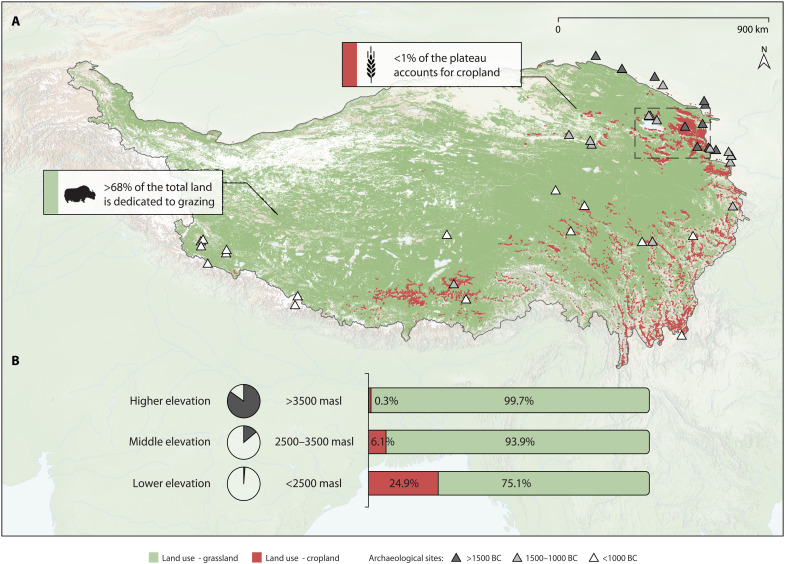
The distribution of modern land use on the Tibetan Plateau. (**A**) Map shows the distribution of modern grassland/pasture (green) and cropland (red) (text S1). The dashed square shows the study region of the barley hypothesis paper (*9*). Triangles display published sites with domesticated animal remains for different time periods (dataset S1). (**B**) Left: Percentage of total land area constituted by different elevation ranges on the plateau. Right: Relative proportion of modern cropland and grassland at different elevations of the plateau (table S1).

Previous research suggests that crop cultivation based on frost-tolerant barley was one of the critical adaptative strategies enabling more sustained habitation of the Tibetan Plateau, facilitating its permanent occupation by around 3600 years ago (*9*). However, this finding reflects data collected only from arable regions of the plateau situated below 3500 m above sea level (masl) (Fig. 1A). These lower-elevation (<2500 masl) and middle-elevation (2500 to 3500 masl) regions account for just ~15% of the total land area of the Tibetan Plateau, yet accommodate more than 71% of its modern farmland (Fig. 1, text S1, and table S1). In the remaining higher-elevation regions (>3500 masl), which constitute ~85% of Tibet’s land area, only 0.2% of land is arable (Fig. 1 and table S1). Archaeologists have found evidence of successful barley farming in these higher-elevation regions, but only in limited warm valleys (*10*–*14*). How prehistoric populations adapted to the remainder of the vast, agriculturally poor highlands of the Tibetan Plateau remains an open question.

Today, 68% of the Tibetan Plateau’s area is dedicated to grazing, while farmland accounts for less than 1% of total land (Fig. 1). Accordingly, mobile pastoralism as well as diverse patterns of agropastoralism (*15*) are key to habitation of the plateau’s higher and more extreme environments, making the Tibetan Plateau home to one of the world’s largest pastoral ecosystems (*16*). High altitude–adapted ruminants (*17*) produce not only meat, hair, and wool for clothing and tents, and fuel from dung, but also milk and processed dairy products that—as renewable, nutrient-rich, storable, and portable foods—contribute significantly to the dietary calories of Tibetan populations (*18*–*20*).

Globally, dairy-based pastoralism was crucial for increasing the life expectancy and population size of prehistoric groups in many regions. The earliest documented dairying to date is in Anatolia (~6500 BCE) (*21*), from where the practice of milking animals appears to have spread rapidly into Europe (~6000 BCE) (*22*) and Africa (~4000 BCE) (*23*). Dairy pastoralism also spread eastward and contributed to ancient demographic success in Mongolia (~3000 BCE) (*24*–*26*) and Xinjiang (~1800 BCE) (*27*–*29*), where productive farming is challenging. According to ancient Chinese sources (e.g., *Tongdian*, 766 to 801 CE; *Old Book of Tang*, 941 to 945 CE), Tibetans have relied on dairy products from cattle/yak and sheep/goat since at least the mid-first millennium CE. Despite the centrality of dairy pastoralism in this region, however, its emergence on the highland Tibetan Plateau remains obscure.

While faunal remains and slaughter patterns can indirectly hint at the possibility of milk consumption, very limited zooarchaeological research has been conducted to date on the interior Tibetan Plateau, especially for earlier time periods (Fig. 1A and dataset S1). Previously published zooarchaeological studies suggest that pastoralism appeared on the highland plateau by around ~3500 years ago (dataset S1). Although “kill-off patterns” can be drawn upon to examine milk exploitation (*30*), the majority of domesticated ruminant bones in Tibet were collected from burial sites rather than settlements, leaving minimal data from which to reconstruct animal management systems or dairying. While it is often assumed that pastoralists inevitably milk their animals, ethnographic studies suggest otherwise (*20*, *31*). Some pastoralists exploit their animals primarily for meat, traction, or fur, while others with mixed herds choose to milk certain species and not others (*20*, *31*–*33*). Accordingly, to better understand the origins and spread of dairying, recovery of direct evidence of ancient milk consumption is critical.

**Fig. 2.**
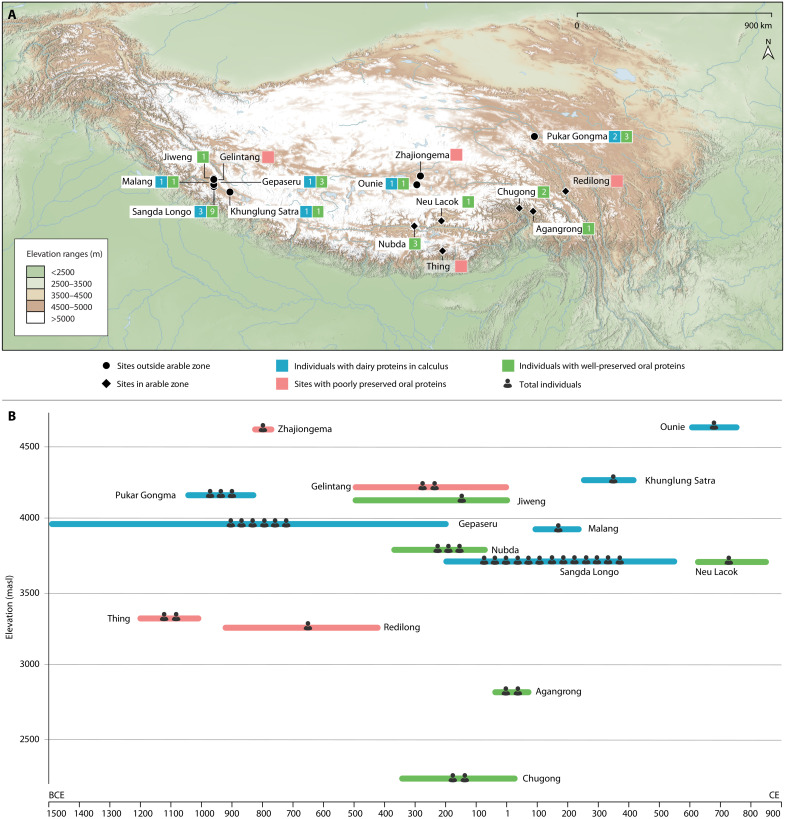
Map, chronology, and elevation of samples included in this study and protein results for studied individuals. (**A**) Distribution of sites investigated in the present study and preservation of milk and oral proteins for individuals from those sites (table S2 and datasets S2 and S3). (**B**) Timeline and elevation of studied samples, total number of human individuals from each site, and protein preservation and dairy recovery from their calculus (text S2 and dataset S2).

To investigate the antiquity of milk consumption on the Tibetan Plateau, we analyzed proteins from ancient dental calculus across the interior regions, dating, where relevant, associated human remains (Fig. 2, Materials and Methods, text S2, and table S2). Dental calculus provides a direct source of ancient dietary information because food proteins and other substances become trapped in the calcified matrix during its formation, offering, in some cases, a context for long-term biomolecular preservation (text S3) (*34*, *35*). In addition to providing dietary information at the level of the individual, species differences in the amino acid sequences of some milk proteins allow preserved proteins to shed light on the species or genus of the animal(s) whose milk was consumed through comparison of recovered sequences to databases of known proteins (*34*). Our research suggests that dairy pastoralism was adopted on the interior plateau by at least 3500 years ago, with sustainable dairy resources from sheep, goat, and possibly cattle/yak supporting pastoralists as they expanded onto the extensive agriculturally poor steppes of the Tibetan Plateau.

## RESULTS

We analyzed dental calculus from 40 human individuals from 15 ecologically diverse sites in Tibet and western Qinghai (text S2 and table S2). Our samples span the late Neolithic through to the Tibetan Imperial periods (ca. 1500 BCE to 842 CE). To further explore the contribution of dairy pastoralism in different landscapes, we divided our samples into two groups, based on whether they derived from arable or non-arable regions, according to the relative proportion of modern cropland (text S1 and table S3). Most individuals—a total of 29 across nine sites—originate from the agriculturally poor regions of the western and northern Tibetan Plateau (Fig. 2). A total of 11 individuals came from six sites located in the more cultivatable regions of the southern plateau. While our sample size is limited, it does include all excavated individuals with sufficient calculus (>5 mg) from within the study area, and accordingly offers as comprehensive a picture of prehistoric dairying on the Tibetan Plateau as is currently possible using dental calculus.

To assess the preservation and possible contamination of the dental calculus proteomes studied, both ancient samples and laboratory controls were screened against the Oral Signature Screening Database (OSSD) (see Materials and Methods, “Protein preservation” section). Of the 40 dental calculus samples analyzed, 26 (65%) displayed well-preserved oral signature proteins, including 19 of 29 individuals (66%) from agriculturally marginal regions, and 7 of 11 individuals (64%) from the plateau’s arable regions (Fig. 2, Materials and Methods, and dataset S2). Samples that passed the OSSD contained oral microbial proteins from multiple groups, including *Actinomyces* sp., *Tannerella forsythia*, and *Porphyromonas gingivalis*, as well as human immune proteins (dataset S3).

No dairy proteins were recovered from the calculus of any of the 7 individuals with well-preserved oral signatures from arable regions, while, in contrast, dairy proteins were identified from 9 of the 19 individuals (47%) from seven sites with well-preserved oral signature proteins from the non-arable highlands (Fig. 2 and datasets S2 and S4). Notably, six of seven (86%) sites in the agriculturally marginal regions that had individuals with well-preserved oral proteins showed evidence for dairy consumption (Fig. 2). A Fisher’s exact test comparing the proportion of individuals with well-preserved oral proteins in the two different landscapes did not show a statistically significant difference (two-tailed *P* = 1). The comparison of the proportion of individuals with dairy proteins in the two types of landscape was significant for the 10% level, with a *P* value slightly above the 5% level (two-tailed *P* = 0.06).

**Fig. 3.**
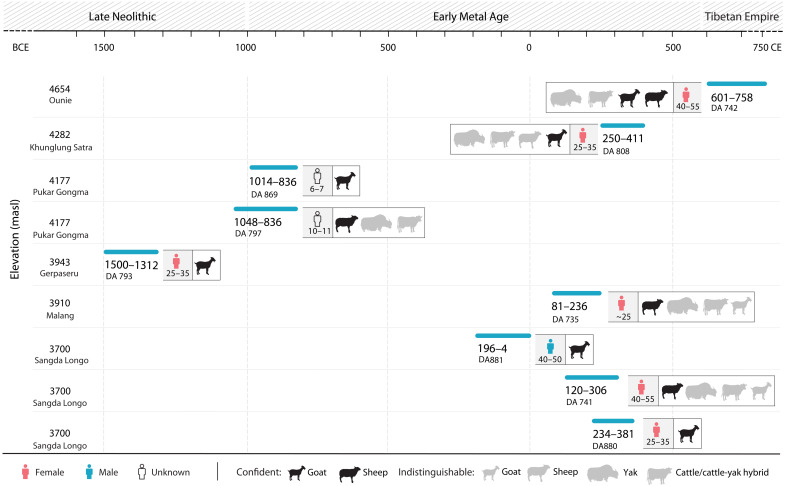
Proteomic evidence of dairy products in dental calculus from the interior Tibetan Plateau. Timeline and altitude of individuals whose calculus produced dairy peptides, together with information about their age, sex, and the taxa identified (confident attribution of animal species shown in black and indistinguishable species shown in gray) (details in Materials and Methods, “Protein identification” section; table S4).

All identified milk peptides derived from the whey protein β-lactoglobulin (BLG) (dataset S4). The dominance of BLG was not unexpected since it is the most commonly reported milk protein from ancient dental calculus (*23*, *25*, *26*, *33*, *34*, *36*–*40*). We observed direct evidence for the consumption of milk from *Ovis* (sheep), *Capra* (goat), and possibly one or more Bovinae species (cattle, yak, and/or their hybrids) (Fig. 3, fig. S1, and dataset S4); the latter taxonomic identification could not be confirmed because of possible deamidation (details in Materials and Methods, “Protein identification” section). For the identified Bovidae milk peptides (*n* = 41), 63% (*n* = 26) of them specifically matched to goat (*n* = 11), sheep (*n* = 8), and sheep/goat (*n* = 7), while the rest (37%, *n* = 15) were assigned to either sheep or Bovinae (fig. S1). We did not detect any milk proteins specifically identified to horse, although horse skeletons were recovered from burials at some of the sites we studied (text S2).

### Widespread evidence for dairy pastoralism in the non-arable highlands

**Fig. 4.**
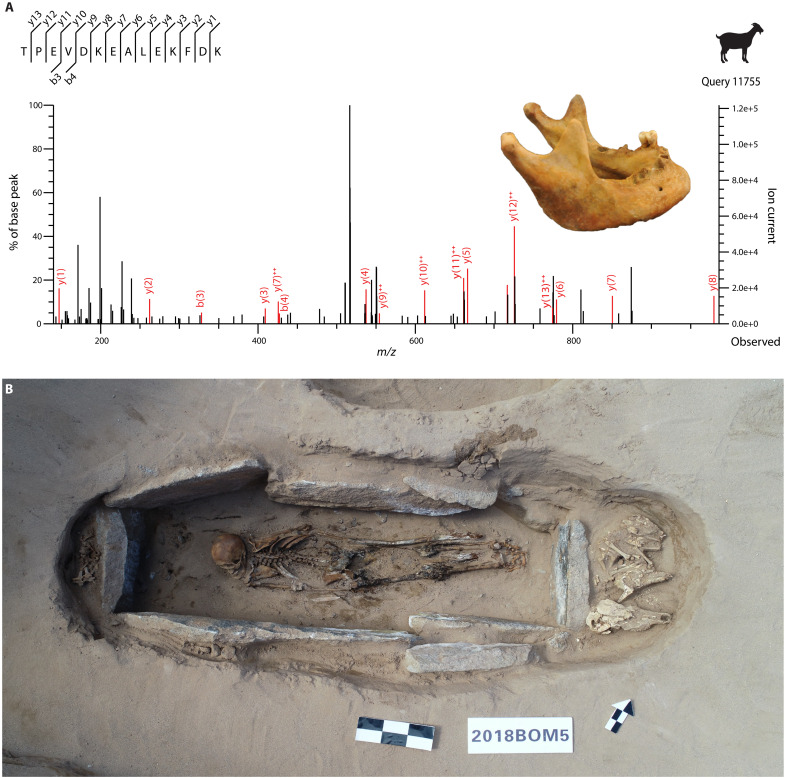
Representative individuals with milk consumption evidence. (**A**) MS/MS spectra of a goat BLG peptide from the earliest individual from whom dairy peptides were recovered (DA793; cal. 1500 to 1312 BCE). (**B**) Highest altitude individual in this study (DA742; cal. 601 to 758 CE; 4654 masl), from the Ounie cemetery in northern Tibet, accompanied by cattle/yak (Bovinae) and sheep/goat (Caprinae) bones (photograph by Z.C.).

To study the emergence of dairying in the agriculturally marginal regions of the Tibetan Plateau, we grouped individuals from the western and northern steppes together (*n* = 29). This included 24 samples from six sites in western Tibet, ranging in date from ~1506 BCE to 537 CE, and in elevation from 3700 to 4282 masl: Gepaseru (*n* = 6), Gelintang (*n* = 2), Jiweng (*n* = 1), Sangda Longo (*n* = 13), Malang (*n* = 1), and Khunglung Satra (*n* = 1) (Fig. 2 and text S2). From these sites, a total of 6 of 15 individuals with well-preserved oral proteins showed evidence for milk consumption (Fig. 2 and table S2). The earliest dairying evidence was recovered from Gepaseru, identified in three of the six tested samples with well-preserved oral proteins. The calculus of one individual from Gepaseru (DA793: cal. 1500 to 1312 BCE) contained BLG peptides specific to *Capra* (Fig. 4A and figs. S2 and S3). Three of the nine well-preserved samples at Sangda Longo (DA881: cal. 198 BCE to 4 CE; DA741: cal. 120 to 306 CE; DA880: cal. 234 to 381 CE) were detected with milk peptides, deriving from *Capra*, *Ovis*, and possible Bovinae BLG (figs. S4 to S7). Dental calculus from Malang (DA735: cal. 81 to 236 CE) contained milk peptides assigned to *Ovis* and the less taxonomically specific Caprinae and possible Bovinae classifications (fig. S8). Milk proteins from Khunglung Satra (DA808: cal. 250 to 411 CE) matched to *Capra*, and possible Bovinae/Ovis (Fig. 3 and fig. S9). Calculus from three further western steppe individuals did not contain any dietary proteins. A single individual from Jiweng with dental calculus with evidence of well-preserved oral-related proteins did not contain any dairy protein evidence. Two additional calculus samples from Gelintang did not pass our preservation threshold (dataset S2).

From the northern highlands, we analyzed five samples from three sites, with dates spanning ~1048 BCE to 758 CE, at elevations ranging from 4177 to 4684 masl: Pukar Gongma (*n* = 3), Zhajiongema (*n* = 1), and Ounie (*n* = 1) (Fig. 2 and text S2). We observed dairy proteins in three of four well-preserved samples (Fig. 3). At the Pukar Gongma site, two of three dental calculus samples with well-preserved oral proteins (DA797: cal. 1048 to 836 BCE; DA869: cal. 1014 to 836 BCE) contained milk peptides derived from possible Bovinae subfamilies, with additional identifications more specifically to *Ovis* and *Capra* (figs. S10 to S12). The dental calculus from Ounie (DA742: cal. 601 to 758 CE) had the largest number of identified milk peptides, also deriving from *Ovis*, *Capra*, and possibly Bovinae (Fig. 4B, fig. S13, and dataset S4). The single sample from Zhajiongema failed to pass preservation screening.

### Lack of evidence for milk consumption in the arable regions

Dental calculus samples from the relatively low elevation regions of the south-central and southeastern parts of the plateau were also studied to explore the antiquity of dairying in more arable regions of Tibet. While 7 of 11 studied samples contained well-preserved oral proteins, none revealed any direct evidence of milk consumption (dataset S2). We studied six samples from three sites on the south-central plateau, ranging from ~1209 BCE to 842 CE and 3314 to 3833 masl: Thing (*n* = 2), Nubda (*n* = 3), and Neu Lacok (*n* = 1) (Fig. 2 and text S2). Neither of the two individuals from the early burial of Thing passed our preservation threshold due to poor overall protein preservation. Three individuals from Nubda and one Tibetan Empire individual from Neu Lacok did contain well-preserved oral proteins but did not contain any milk proteins in their calculus.

Protein extractions were also conducted on five dental calculus samples from three sites in the southeastern plateau, dating from ~926 BCE to 75 CE, at elevations from 2243 to 3260 masl: Redilong (*n* = 1), Chugong (*n* = 2), and Agangrong (*n* = 2) (Fig. 2 and text S2). The dental calculus from Redilong contained an extremely small quantity of protein, making dietary analysis impossible. One of two samples from Agangrong, and both samples from Chugong, retained well-preserved oral signatures but again lacked milk proteins (datasets S2 and S3).

## DISCUSSION

While cold-tolerant barley was undoubtedly important for establishing long-term settlement in the arable valleys of the Tibetan Plateau, as suggested by previous research (*9*–*14*), the cold and arid environment of the vast majority of the higher-elevation plateau is too extreme for productive barley farming (Fig. 1). Understanding how ancient populations obtained sufficient food to survive in these agriculturally marginal highlands is key to understanding the long-term economic, demographic, and land-use development of the Tibetan Plateau. We identified milk proteins from the dental calculus of nine highland individuals, providing the earliest direct evidence of ancient dairy consumption on the plateau. In addition, we observed a highly contrasting pattern of milk protein recovery in different ecological niches, with all our recovered milk peptides coming from ancient individuals in the non-arable steppes rather than the arable valleys. Our research suggests that dairying supported early pastoralist occupation of the non-arable highland plateau, a vast region where agriculture is challenging, but ruminants could convert the energy locked in alpine pastures into nutritional milk and meat. Dairying appears to have been a critical cultural adaptation that allowed Late Holocene humans to optimize exploitation of the natural resources on the highland Tibetan Plateau.

Our findings indicate that dairying was introduced onto the interior Tibetan Plateau by at least 3500 years ago, more than 2000 years earlier than recorded in historical sources (*Tongdian*, 766 to 801 CE). This timing coincides with the earliest archaeologically identified domesticated ruminant bones on the interior plateau, indicating that dairying was probably adopted as soon as pastoralism spread into this region. The emergence of dairying on the highland plateau could potentially trace back at least 200 years earlier than this, as the earliest individual (2017ZGM8) at Gepaseru, where we obtained the oldest milk proteins, has been securely dated to cal. 1883 to 1691 BCE (GU55772; 3464 ± 29). Unfortunately, insufficient dental calculus preservation made it impossible to further investigate milk consumption in this early individual through proteomics, although the discovery of sheep and goat skulls near the human remains (*41*) suggested that these species were potentially milked by ~3800 years ago. Further work is needed, ideally combining proteomics with zooarchaeological, lipid, and paleoecological research, to clarify how early both dairying and pastoralism can be traced back on the Tibetan Plateau.

Our paleoproteomic data further suggest that dairying played a more significant role in supporting pastoralist expansions into the less arable western and northern highlands, from where all our milk proteins were recovered, than the more arable valleys on the southern plateau (Fig. 2). The net primary productivity of the western and northern plateau is relatively low due to the dominance of high-cold steppe (*42*), and modern populations living in these highlands rely heavily on pastoral resources. Ethnographic surveys in Tibet indicate that highland herder subsistence has traditionally focused on dairy products (e.g., milk, butter, cheese, and yogurt), mainly because consumption of meat requires killing of livestock and usually occurs only in a specific season (*18*, *19*). Milk and milk products provide a sustainable and renewable food source that contributes to increased food security in the Tibetan highlands. Our data show that dairy products supported highland pastoralists by as early as the mid-second millennium BCE. Four of the nine individuals with evidence for milk consumption were located above 4000 masl, with the highest located at the extreme height of 4654 masl (Figs. 3 and 4B). These are the highest altitude ancient dairying findings reported anywhere in the world to date. Up to 80% of sites (four of five) in western Tibet and 100% of sites (two of two) in the northern plateau with well-preserved samples show direct milk consumption evidence, indicating that dairying was not uncommon in these non-arable steppic regions in prehistory.

In contrast, we did not detect any milk proteins from more arable region samples in southern-central and south-eastern Tibet, despite analyzing all ancient human individuals with sufficient dental calculus from these regions. This pattern suggests that dairy consumption may have been less critical in the plateau’s arable valleys than that in its agriculturally poor highlands. While 7 of the 11 individuals from four sites in the southern plateau contained well-preserved oral proteins, none of them showed reliable evidence of milk proteins (Fig. 2 and dataset S2). An absence of recovered dietary proteins does not, however, mean that dairy foods were not consumed (text S3). It is possible that dairy products were consumed but played a relatively minor role in the diets of individuals living in the valleys, where grains may have contributed more significantly to human calories. It is alternatively possible that the relative warmer valleys offered more challenging contexts for the preservation of milk proteins than the cooler highlands, shaping the patterns we observed in our dataset. Further research is accordingly required, particularly on calculus from early skeletal remains excavated in the future, as well as through parallel studies of lipids potentially preserved in ancient ceramics, to explore the exact timing of dairying initiation in the arable regions of the Tibetan Plateau.

In regions where milking was practiced, dairying appears to have provided nutrition to individuals across a broad gender, status, and age spectrum (Fig. 3). Dairy was consumed by both females and males, as well as in individuals from both elite and non-elite burial contexts. Elite burials like the tomb cluster of Sangda Longo (text S2 and fig. S4), with its abundance of high-value artifacts like gold masks and bronze weapons, showed evidence for dairy consumption, but so too did individuals from non-elite burials, such as Gepaseru, Pukar Gongma, and Malang. While most milk protein identifications came from adult individuals (Fig. 3 and table S4), we were also able to identify goat milk peptides from one 6/7-year-old individual (DA869; cal. 1014 to 836 BCE) at Pukar Gongma (Fig. 3 and fig. S10), suggesting that ruminant milk prolonged the benefits of balanced nutrition that children obtained from breastfeeding. These findings are significant because while lipid evidence has been used to indirectly suggest that animal milk was used as a supplementary food for children in Neolithic Europe (*43*), milk proteins have not previously been reported from the dental calculus of children in Europe or indeed anywhere else, as calculus is rarely recovered from juvenile dentition. The consumption of dairy by groups of variable age and status, especially those with a limited ability to access other nutritional foods, likely played a vital role in boosting highland population growth over time.

In addition to demonstrating that milk was consumed by diverse populations, our paleoproteomic data also suggest that a range of dairy livestock was important on the early highland plateau (Fig. 3). While various limitations prevent us from drawing detailed conclusions from the species data, direct evidence for the ancient milking of goat, sheep, and possibly Bovinae species was also obtained. Given that goat BLG can be more challenging to detect by liquid chromatography–tandem mass spectrometry (LC-MS/MS) (*44*), the dominance of goat-specific peptides in our samples would potentially suggest a preference for goat milk on the early western plateau. One animal for which no direct dairying evidence could be confirmed, surprisingly, was the yak. Yaks have numerous anatomical and physiological features that equip them for life at high altitude (*45*), and their milk is richer in proteins, fats, and bioactive compounds than the milk of other ruminant species (*46*), making them critical for maintaining food security in the highlands today. While the adoption of yak pastoralism has accordingly long been recognized as one of the most important developments supporting early human occupation of the highland plateau, we currently lack robust, definitive proteomic evidence in support of the milking of this species. However, this limitation likely relates to the fact that differentiating between cattle and yak whey protein (BLG) is challenging because only a single amino acid difference distinguishes the BLG sequence of the two species (*47*). This makes it impossible to differentiate between the two species if the diagnostic amino acid sequence is not recovered, meaning that we recovered possible Bovinae peptides from multiple individuals, but could not assign them to a more specific taxonomic group. Further testing of calculus samples from the Tibetan Plateau may confirm the presence of cattle and/or yak milk consumption from an early date.

Dramatically increased numbers of settlement and burial sites after ~1500 BCE on the highland Tibetan Plateau indicate that the adoption of dairy pastoralism, together with barley farming in warm valleys, helped to revolutionize human occupation and land-use patterns in this region. Research indicates that dairy pastoralism offers a more efficient way of converting natural resources to human energy than hunting (*48*). While the exploitation of wild plants and animals supported the presence of archaic hominins living in the lower margins of the plateau by at least ~160,000 years ago (*49*–*52*) (and probably allowed them to reach the interior plateau by ~226,000 years ago) (*53*), and *Homo sapiens* had begun to explore the interior plateau by ~40,000 to 30,000 years ago (*54*), there is a general consensus that the special challenges of the highland plateau limited year-round occupation on a significant scale (*55*). The spread of millet farming, in combination with the continued exploitation of local wild resources, enabled more inhabitants to settle in limited ecologically rich pockets of the high-elevation valleys after ~3200 BCE (*9*, *56*–*58*), while barley cultivation pushed these limits from the mid-second millennium BCE, enabling expansion of human occupation into much more higher-altitude valleys (*9**–**13*). Nonetheless, vast non-arable regions of the plateau likely remained challenging for long-term and year-round occupation until the adoption of mobile pastoralism (*55*). Our findings suggest that pastoralism, particularly linked to the exploitation of portable and renewable dairy resources, helped to make larger-scale habitation of the Tibetan Plateau’s extensive non-arable highlands possible.

As a flexible strategy enabling mobility and optimizing exploitation of the plateau’s limited resources, dairy pastoralism would also have offered a powerful cultural adaptation for weathering several waves of cooling and aridification on the Tibetan Plateau after the early Holocene (*59*). The combination of dairy pastoralism and barley farming, which enabled much more effective exploitation of high-altitude resources than any previous subsistence lifeway, would likely have been equally essential to prehistoric demographic success on the plateau, and the establishment of the Tibetan Empire and other regional kingdoms. While this multi-millennia-old adaptation may have made ancient agropastoralists and mobile pastoralists on the plateau better adapted to hyper-cold environments and more resilient to climate cooling, how it fairs moving into the 21st century under the effects of global warming remains to be seen (*60*).

## MATERIALS AND METHODS

### Dental calculus collection

To examine ancient dairy consumption history on the Tibetan Plateau, we analyzed ancient proteins extracted from the dental calculus of 40 individuals excavated from Tibet and western Qinghai. We selected all the human individuals with sufficient dental calculus (>5 mg) from 15 archaeological sites on the interior Tibetan Plateau to achieve broad geographic and chronological coverage. The detailed archaeological contexts for each site are described in text S2. Sampling was conducted in a clean space. Dental calculus was removed from the teeth using disposable scalpels and transferred to aluminum foil, then sealed and stored in plastic sampling bags. Nitrile gloves, medical masks, and hair nets were used during sampling to reduce the potential for modern contamination. Scalpels, gloves, and the sampling surface were carefully cleaned with 70% alcohol wipes between the sampling of different individuals to avoid cross-sample contamination.

### Protein extraction and LC-MS/MS

To avoid contamination with modern material, protein extractions were conducted in a dedicated clean laboratory used only for extracting archaeological proteins. No modern samples are stored or extracted in the laboratory, and the extraction positive control is a well-preserved archaeological bone. Clean laboratory-specific garments, as well as hair nets, face masks, and gloves, are worn at all times. Furthermore, all tools and surfaces are cleaned with bleach, milliQ water, and ethanol before each extraction.

We separated the ancient dental calculus into five extraction batches; for each batch, we added one extraction blank as a negative control (“Blank”) and one ancient sheep bone powder sample as a positive control (“Bone control”). In total, 40 dental calculus samples, 5 blanks, and 5 bone controls were extracted (dataset S2). All the samples were extracted using a published modified single-pot, solid phase–enhanced sample preparation (SP3) protocol (*23*, *61*), which can also be found on protocols.io DOI: dx.doi.org/10.17504/protocols.io.bfgrjjv6. Briefly, samples were demineralized with Ethylenediaminetetraacetic acid (EDTA) (0.5 M, pH 8) for 4 to 7 days, and then denatured with GuHCl, reduced with Tris(2-carboxyethyl)phosphine (TCEP), and alkylated with chloroacetamide (CAA). We collected the proteins using Magnetic Speedbeads followed by digestion with trypsin for 18 hours at 37°C. Peptides were desalted with C18 StageTips. Samples were then transported to the Functional Genomics Center Zürich, ETH/University of Zürich for LC-MS/MS analysis. LC-MS/MS analysis was performed on a Q-Exactive spectrometer (Thermo Fisher Scientific, Bremen, Germany) equipped with a Digital PicoView source (New Objective) and coupled to a nanoACQUITY UPLC (Waters AG, Baden-Dättwil, Switzerland). Solvent composition at the two channels was 0.1% formic acid for channel A and 0.1% formic acid, 99.9% acetonitrile for channel B. Column temperature was set to 50°C. For each sample, 4 μl of peptides was loaded on a commercial Symmetry C18 Trap Column (100 Å, 5 μm, 180 μm × 20 mm; 2G, V/M, Waters) followed by a C18 HSS T3 Column (100 Å, 1.8 μm, 75 μm × 150 mm, Waters). The peptides were eluted at a flow rate of 300 nl/min by a gradient from 8 to 22% B in 49 min and 32% B in 11 min. We monitored potential sample carryover through the use of injection blanks between each sample. After each run, the column was cleaned with 95% solvent B for 5 min before reestablishing the loading condition.

Mass spectrometers were operated in data-dependent mode performing higher-energy collision dissociation (HCD) fragmentation on the 12 most intense signals per cycle. The full-scan MS spectra [300 to 1700 mass-to-charge ratio (*m/z*)] were acquired at a resolution of 70,000 at 200 *m/z* after accumulation of a target value of 3 × 10^6^. HCD spectra were acquired at a resolution of 35,000 using a normalized collision energy of 25 and a maximum injection time of 110 ms. The automatic gain control was set to 5 × 10^4^ ions. Charge state screening was enabled. Singly, unassigned, and charge states higher than seven were rejected. Only precursors with an intensity above 9.1 × 10^3^ were selected for MS/MS measurement.

### Protein database searching

Tandem mass spectra raw files were converted to Mascot generic files in MSConvert (v.3.0.11781) using the 100 most intense peaks from each spectrum. The transformed MS/MS data were searched with Mascot (Matrix Science; v.2.6.0) software using the SwissProt (v.2020_04; 561,911 sequences) database in combination with a custom dairy database consisting of 245 dairy livestock milk protein sequences, both reviewed and unreviewed, obtained from the UniProt and TrEMBL databases (*26*). Mascot searches were conducted with a peptide mass tolerance of 10 parts per million and fragment ions mass tolerance of 0.01 Da. Up to three missed cleavages were allowed. Carbamidomethyl of cysteine was set as a fixed modification. We ran the error tolerance of four samples (DA741, DA742, DA793, and DA797) that contained the most abundant milk peptides and found that oxidation of methionine and the deamidation of both asparagine and glutamine were the only common posttranslational modifications; therefore, they were set as variable modifications. From the results of this test, we included these variable modifications in our searches. The ion score significance threshold was set to *P* < 0.05 for our initial Mascot searches before data filtering in Scaffold.

### Protein authentication

#### Protein identification

Proteins were filtered in Scaffold (v.4.11.0) with the following criteria: (i) protein false discovery rate (FDR) less than 1% and peptide FDR less than 1%; (ii) protein identification probability higher than 95% and, in addition, each individual peptide identification probability higher than 85%; (iii) each protein was supported by a minimum of two unique peptide spectral matches (PSMs). All the PSMs assigned to dietary proteins were searched using BLASTp, which compared the sequences against the National Center for Biotechnology Information (NCBI) nonredundant database and identified all species to which sequences matched (*25*).

For the BLG peptides we obtained (dataset S4), the variation in amino acid sequence (positions 143 to 156) TPEVD(N/K/D)EALEKFDK enables us to distinguish between Bovinae, *Capra*, and *Ovis*. If we detect an asparagine (N) at position 148, the sample we analyzed contains *Ovis* milk (“confident sheep”), and a lysine (K) uniquely assigns to *Capra* milk (“confident goat”). Theoretically, an aspartic acid (D) is specific to Bovinae. However, the presence of a D at position 148 does not enable a unique assignation to Bovinae as a common taphonomic modification in archaeological samples is the deamidation of N to D and glutamine (Q) to glutamic acid (E). It is impossible to distinguish a deamidated N from an unmodified D via LC-MS/MS, since they have identical masses. We therefore classify peptides with a D at position 148 into the broad category of *Ovis*/Bovinae (“indistinguishable sheep, cattle, yak, or cattle-yak hybrid”). In addition, K is a tryptic cut site and the diagnostic peptide for goat (TPEVDK) is too short for detection without a missed cleavage, biasing against the detection of goat milk (*44*). Other peptide sequences, such as VYVEELKPTPEGNLEILLQK and LAFNPTQLEGQCHV, are conserved across the subfamily Caprinae, with sheep and goat accordingly indistinguishable in these cases (“indistinguishable goat, sheep” group).

#### Protein preservation

To evaluate the preservation of the overall dental calculus sample proteome, we assessed the presence of proteins that would be commonly found in a human oral cavity. To do this, we ran our data against the published OSSD (*23*). The OSSD contains common contamination proteins, including those encountered in the environment and laboratory, as well as oral microbe and human immune response proteins. The screening method relies on the assumption that well-preserved ancient dental calculus will encapsulate a range of oral bacteria and immune proteins commonly found in saliva. Therefore, dietary proteins from calculus samples with an oral signature (i.e., that pass the OSSD threshold) have a higher potential to offer reliable ancient diet information. To pass the OSSD threshold, individuals required a total of at least 15 OSSD proteins, of which more than 65% were oral bacteria and/or human inflammatory response proteins. In total, samples from the calculus of 26 of the 40 examined ancient individuals passed the OSSD threshold, including all samples in which milk peptides were detected. Of the dental calculus samples that failed to pass the OSSD threshold, none contained dairy proteins. In addition, none of the blank and bone control samples passed the OSSD filtering threshold (datasets S2 and S3).

Dairy proteins were identified in nine individuals with well- preserved oral signatures. Deamidation of dairy peptides [TPEVD(N)EALEK] was assessed, excluding the location where deamidation is indistinguishable from a known amino acid difference (D/N). Of the nine milk-positive individuals, five had only dairy peptides, which contained no deamidation locations or locations that could not be assessed (D/N known amino acid variation). Two individuals only had one peptide with one deamidation site each, which is insufficient for analysis; therefore, the deamidation rates were not calculated. The other two individuals show over 40% deamidation of assessable deamidation sites in dairy peptides (11 of 16 in DA741 and 11 of 26 in DA742). These results indicate that there is no reason to conclude that the dairy peptides are not authentic.

### Radiocarbon dating

A total of 19 human bone samples were sent for collagen radiocarbon dating in this study; 18 of them were dated at Beta Analytic Testing Laboratory, Miami (laboratory code: BETA), and 1 sample was analyzed at Scottish Universities Environmental Research Centre Radiocarbon Laboratory Glasgow (laboratory code: SUERC). Bone collagen was extracted at BETA using an acid step for bone demineralization and an alkali step to remove soil humics (with alkali, Beta Analytic 2021, www.radiocarbon.com/ams-dating-bones.htm#Components). At SUERC, one bone sample was mechanically cleaned and subjected to an acid-alkali-acid chemical protocol followed by solubilization, filtration, and lyophilization before Accelerator Mass Spectrometry (AMS) ^14^C dating (*62*). All radiocarbon measurement results were calibrated using the calibration software OxCal v.4.4.4 (*63*) and the IntCal20 radiocarbon calibration curve for the northern hemisphere (*64*) (see text S2 and table S4).

### Statistical analysis

Given the small sample size, we used a Fisher’s exact test to compare sample preservation and the presence of dairy consumption. We assessed the statistical significance for 5 and 10% levels.

## Supplementary Materials

### This PDF file includes:

Texts S1 to S3 Figs. S1 to S13 Tables S1 to S4 Legends for datasets S1 to S4 References

### Other Supplementary Material for this manuscript includes the following:

Datasets S1 to S4
