# Supplementary material for: Paleoproteomic evidence reveals dairying supported prehistoric occupation of the highland Tibetan Plateau
Source: Sci Adv. 2023 Apr 12;9(15):eadf0345. doi: 10.1126/sciadv.adf0345 (PMC10096579; doi:10.1126/sciadv.adf0345)
Supplement: Supplementary file 1 — Texts S1 to S3 Figs. S1 to S13 Tables S1 to S4 Legends for datasets S1 to S4 References [file sciadv.adf0345_sm.pdf]

Science Advances
AAAS

Supplementary Materials for

Paleoproteomic evidence reveals dairying supported prehistoric occupation of the highland Tibetan Plateau

Li Tang *et al.*

Corresponding author: Li Tang, tang@shh.mpg.de; Hongliang Lu, luhl@scu.edu.cn; Shargan Wangdue, xiagewangdui@163.com; Nicole Boivin, boivin@shh.mpg.de

Sci. Adv. **9**, eadf0345 (2023)
DOI: 10.1126/sciadv.adf0345

The PDF file includes:

Texts S1 to S3
Figs. S1 to S13
Tables S1 to S4
Legends for datasets S1 to S4
References

Other Supplementary Material for this manuscript includes the following:

Datasets S1 to S4

Text S1: Modern land use on the Tibetan Plateau

Known as the world's largest high-altitude landmass, the Tibetan Plateau covers an area of 2,542,298 km^2. The vast majority of the plateau surface sits at an elevation above 3500 masl, accounting for ~84.73% (2,154,019 km^2) of its total land area (*65,66*). There are only limited pockets (~15.27%; 388,281 km^2), such as river basins, lake terraces, and margins, that sit at lower elevations, including middle-high elevation regions (2500-3500 masl) that account for ~14.03% (356,651 km^2), and low-altitude below 2500 masl that make up ~1.24% (31,630 km^2) of the total territory (table S1). Overall, the average elevation increases from the eastern and central plateau, to its western part (*67*). The entire plateau is primarily characterized by an arid and cold climate (*68*), although rainfall and temperature vary across different regions. The eastern plateau receives the most abundant precipitation as the Indian Summer Monsoon collides into the eastern rim of the Tibetan Plateau, then gradually decreases in intensity as it moves west. Additionally, the eastern plateau also has much higher mean annual temperatures than other regions.

Variable ecology and topography across the Tibetan Plateau shape diverse high-altitude adaptation strategies and modern land-use practices (*15*). A large portion of the plateau (68.42%, 1,739,414 km^2) is only suitable for grazing. The relative proportion of the plateau's agropastoral land that is dedicated to grazing (grassland) increases with altitude (Fig. 1, table S1); ~91.10% of the plateau's total grassland is distributed across higher elevation regions above 3500 masl. In contrast to the extensive grassland, farmland only accounts for ~0.70% (17,716 km^2) of the total land area, and mainly situated in the lower river valleys of the eastern and central-southern plateau where more favorable hydrothermal conditions exist (Fig. 1). Beyond these arable and pasture regions, the remainder of the Tibetan Plateau is dominated by undeveloped land, much of which is covered in glacial ice, surface rock, or hyper-arid desert (*68*).

To study the contribution of dairy pastoralism in different ecological niches, we divided our studied sites into arable and non-arable regions according to the proportion of cropland, and soil quality of arable lands in each city or prefecture (table S3). Three regions (Ngari, Nagqu, and Yushu) contain extreme low proportion of cropland, moreover, possess the poorest soil quality for farming; we therefore sorted them into non-arable regions. The rest of 5 regions, including Qamdo, Nyingchi, Shannan, Xigazi, and Lhasa in the southern plateau, is divided into arable regions.

All the calculations mentioned above are based on the intersection of a digital elevation model in 7.5-arc-second (~250 m) resolution (SRTM 3, CGIAR-CSI Version 4.1) (*69*). The boundary and definition of the Tibetan Plateau followed by Zhang et al. (2014) (*65*). Cropland and grassland data are taken from the land use classification dataset of the Tibetan Plateau (version 1.0, recorded in 1992) (*66*); while this version was recorded around three-decades ago could not reveal the updated land-use, it is the most recent resource that has been released to the public.

Text S2: Archaeological context of studied individuals

The prehistoric archaeology of the interior Tibetan Plateau is often roughly divided into three periods: Palaeolithic (before 5500 BCE), Neolithic (5500-1000 BCE), and Early Metal Age (1000 BCE- ~ 600 CE) (*70–73*). The term Metal Age is used because the scarcity of metal objects from clear archaeological contexts makes it extremely challenging to differentiate between Bronze and Iron Age sites (*70*). The Historic period began after the establishment of the Tibetan Empire (618-842 CE).

We note that excavations on the Tibetan Plateau show both chronological and geographical biases. Across the plateau, 11 Neolithic sites have been discovered and most of them are settlements. Only three Neolithic burial sites have been uncovered, and all of them correspond to the Late Neolithic after 3000 BCE. However, for the Early Metal Age, approximately 36 sites have been excavated, with cemeteries comprising the majority of sites (n=30). The most intensively excavated region containing sites dating to the Early Metal Age is western Tibet, which possesses numerous Early Metal Age burials, often of high-status individuals and/or concentrated in clusters.

To create a comprehensive picture of ancient dairy consumption in the interior Tibetan Plateau, we sampled dental calculus from all of the excavated individuals from Tibet and western Qinghai. In total, we collected samples from four Neolithic individuals, and 36 Early Metal Age individuals from 15 sites (table S2 and dataset S2). The majority of excavated individuals (n=24) are from western Tibet, followed by central Tibet (n=6), eastern Tibet (n=5), western Qinghai (n=3), and northern Tibet (n=2). In the following section, we describe the archaeological context of each studied individual. We use bolded text to highlight individuals that had calculus containing identified milk proteins.

<u>Late Neolithic and Early Metal Age sites in western Tibet</u>

Gepaseru (3943 masl)

Gepaseru is the earliest burial site located in the interior Tibetan Plateau. The site was first discovered in 1997 (*74*), and it was recently excavated over four seasons from 2017 to 2020. In total, 21 burials were excavated, and archaeologists identified two main phases. An earlier phase (~1800-1000 BCE) falls into the Late Neolithic with stone-cist burials accompanied by painted pottery, lithics, ruminant bones, bronze items, and beads. In contrast, the later phase (~400-1 BCE), corresponding to the Early Metal Age, was dominated by shaft tombs containing abundant pottery, ruminant bones, bronze and iron weapons, ornaments, and wooden containers. The associated artefacts and burial practices during the early period are unique, and have not been reported previously, while the material culture for the later period shows similarities with findings across the western Himalayans.

Ancient populations at this site engaged in a herd-based agropastoral economy since ~3800 years ago, they grazed mixed herds containing cattle, sheep, goat, and horse, crop cultivation and hunting

were also practiced as minor subsistence strategies (*41*). Seven individuals from Gepaseru were sampled in this study:

- **2017ZGM2 (DA793; cal. 1500-1312 BCE, GU55771)** is a 25-35-year-old female, and the earliest studied individual in this paper (fig. S2). Cattle, goat, and barley remains in this burial appear to represent the earliest archaeological evidence for these species in western Tibet.
- 2019ZGM6 (DA736; cal. 1506-1407 BCE, Beta-543669) is an individual aged to 28-36 years; the poor preservation of the skeleton did not allow for sex identification.
- 2017ZGM10 (DA800; cal. 368-165 BCE, Beta-486439) is likely a male aged 36-40 years.
- 2019ZGM7 (DA871) is a grave of a possible female aged 39-43.
- 2018ZGM1 (DA872; cal. 385-197 BCE, Beta-513163) is a potential female, approximately 36-40 years of age.
- 2019ZGM4 (DA873) is the grave of a 41-50-year-old female individual who was accompanied by the most abundant artefacts of any burial at the site; this also included remarkable cattle, sheep, and goat heads as part of a sacrifice.

Khunglung Satra (4282 masl)

The Khunglung Satra cemetery was discovered by the Shannxi Academy of Archaeology, and more than 10 stone-cist burials were uncovered from 2017-2019. Human skeletons dated to two phases: an early phase spanning ~700-100 BCE, and a late phase covering ~ 100-400 CE. Only one skeleton from the late phase of this site contained adequate dental calculus for proteomic extraction.

- **2019QLSZM1 (DA808; cal. 250-411 CE, Beta-575047)** is a female who died around the age of 25-35 years. Abundant grave goods were recovered including iron, bronze, wooden artifacts, and textiles. Sheep and cattle bones were also recovered.

Gelintang (4200 masl)

The site of Gelintang is situated alongside the Dungkar River. Ten burials and one horse-sacrifice pit were excavated in 1999. The human remains from the site were dated to ~500-1 BCE. While the human skeletons were poorly preserved, a large number of ceramic vessels, and some bronze and iron artefacts, as well as animal bones, were collected. Only two individuals possessed sufficient dental calculus for protein extraction.

- 1999ZPGM6 (DA799) is a possible male, aged 48-57 years, buried in a single-room shaft tomb. Approximately 17 sheep/goat heads were placed around the burial walls. Additionally, a bronze sword and some seeds (no species information published so far) were also buried next to the human skeletal remains (*75*).
- 1999ZPGM7 (DA870) is a 48-57-year-old male, who was buried with limited grave goods, including one stone spindle whorl, one sheep/goat horn, and some wooden fragments (*75*).

Jiweng (4085 masl)

The cemetery cluster of Jiweng is situated on a second terrace, near the modern agro-pastoral village of Phiyang. Archaeologists excavated 14 burials in 2018. Human remains from large shaft tombs dated to ~500-1 BCE, while those from small burials dated to around 500-1000 CE. While the human skeletons were well preserved, only a few individuals contained adequate dental calculus. We only sampled one individual for this study.

- 2018ZPJM7 (DA877) is a 45-50-year-old male, accompanied by a lacquered wooden plate, bronze and iron weapons, and textiles.

Sangda Longo (3700 masl)

The large-scale burial cluster of Sangda Longo is located on a modern pasture on the northern bank of the Langqên Zangbo (Upper Sutlej River) (fig. S4). The site was densely occupied for approximately 1000 years, from ~350 BCE to 650 CE. Four excavation seasons (2017-2020) uncovered up to 50 burials. A considerable wealth of artefacts was collected from several mass graves, including golden ornaments, beads, textiles, bronze and iron objects, as well as wooden figurines. The excavators assigned these burials to three occupation phases. Phase I (366-200 BCE) saw the emergence of social inequality, with the quantity of high-value items varying across different burials. Phase II (200 BCE- 600 CE) appears to reflect an intensification of social stratification. Archaeologists suggest that some elites in this period controlled significant wealth as well as wider trade networks, as evidenced by the abundance of high-ranking symbolic artefacts associated with this phase. Phase III (600-668 CE) shows a dramatic decline in grave goods and the number of burials, perhaps reflecting a decrease in population size. In total, 13 individuals from this site contained sufficient calculus for proteomics analysis.

- **2018ZSEM3 (DA880; cal. 234-381 CE, Beta-513147)** is a female who died at an estimated age of 25-35 years. This burial contained the most abundant pottery of any grave in this cluster.
- **2018ZSWM3 (DA881; cal. 196 BCE- 4 CE, Beta-513146)** is a male aged 40-50 years.
- **2019ZSEM28 (DA741; cal. 120-306 CE, Beta-543653)** is a female of around 40-55 years of age.
- 2017ZSM6 (DA804) is a female, who died at the age of 39-43 years.
- 2017ZSM7 (DA885) is a juvenile aged 13-14 years; no clear sex characteristics could be observed.
- 2018ZSM5 (DA882) is a 13-14-year-old individual who has ambiguous sex characteristics.
- 2019ZSEM25 (DA883; cal. 262-537 CE, Beta-543631) died at approximately 39-43 years.
- 2019ZSEM31 (DA884) is a male of around 39-43 years of age.
- 2019ZSEM26: 129 (DA805) is a female, who died at around 39-43 years.
- 2019ZSEM26: 179 (DA886) is a 39-43-year-old female.
- 2019ZSEM26: 182 (DA806) is a 6-7-year-old child with ambiguous sex characteristics.
- 2019ZSEM26: 184 (DA887) contained an elderly individual aged over 55 years.
- 2019ZSEM26: 211 (DA888; cal. 425-565 CE, Beta-543635) is a female who died at an estimated age of 30 years.

Malang (3910 masl)

Malang is a cemetery situated on the southern bank of the Langqên Zangbo River. A single cave tomb was discovered in 2019 by local villagers. While tomb passages were destroyed, the chamber contained the original deposits. Remarkable artefacts were collected from the cemetery, including painted pottery, bronze containers and ornaments, iron weapons, wooden and shell ornaments, beads, and textiles. Abundant animal sacrifices (sheep, goat, and horse) were practiced, evidenced mainly by sheep/goat heads. In total, 6 individuals were recovered, but only one of them had sufficient dental calculus.

- **2019ZMM1 (DA735; cal. 81-236 CE, Beta-547974)** is female aged approximately ~25 years. Bronze ornaments and the hair of the individual were well-preserved.

Early Metal Age sites in northern Tibet

Zhajiongema (4648 masl)

The stone-cist burial of Zhajiongema, is located in an extensive pasture in the northeastern part of Siling Co Lake, in northern Tibet. Bronze items, pottery, beads, and sheep/goat bones were buried with a well-preserved individual.

- 2019BZM2 (DA807; cal. 821-769 BCE, Beta-544643) is female individual with an estimated age of 15-20 years.

Ounie (4654 masl)

Ounie is the highest elevation site in this study. The stone-cist burial cluster is situated on a modern pasture to the southeast of Siling Co Lake. More than 20 burials were identified, and five of them were excavated in 2018. Burials 4 and 5, showing better preservation, were accompanied by bronze, iron, ceramic, and bone objects, as well as textiles and beads. Only one individual had enough dental calculus for protein extraction.

- **2018BOM5 (DA742; cal. 601-758 CE, Beta-513157)** is a female approximately 40-55 years of age (Fig. 4B). Cattle/yak and sheep/goat bones were placed in the upper and lower parts of the human skeleton.

Late Neolithic and Early Metal Age sites in southern-central Tibet

Thing (3314 masl)

Thing is the earliest current known burial cluster in central Tibet. Two stone-cist burials were excavated in 2017. The cists contained the largest pottery assemblages yet known in central Tibet, numbering up to 70 complete ceramic vessels. Additionally, a few bronze and stone tools were also recovered. We sampled two individuals for this study:

- 2017LZTCM1-60 (DA794; cal. 1209-1005 BCE, Beta-486440) is an individual with an estimated age of 15 years.
- 2017LZTCM1-64 (DA740) is a female around 20-30 years of age.

Nubda (3833 masl)

Nubda, is located along the midstream portion of the Yarlung Tsangpo River. Four stone-cist burials were exposed in 2019 during roadwork. Human skeletons and ceramics were unearthed, located both inside and outside the tombs. We included three individuals in our study.

- 2019RRNCM1 (DA737; cal. 346-51 BCE, Beta-527361) is a male who died at the age of 35-40 years.
- 2019RRNCM4 (DA875) is a 35-40-year-old male individual.
- 2019RRNCG (DA874; cal. 387-200 BCE, Beta-527362) is aged 28-36 years; no clear sex characteristics can be observed.

Neu Lacok (3700 masl)

Neu Lacok is situated in the city center of Lhasa. In total, 6 burials were recovered in 2003. Excavators suggested that these burials could be attributed to the Tibetan Empire (618-842 CE) based on the characteristics of the burial practices. We sampled one skeleton that had sufficient calculus.

- 2003LWZLJM6 (DA889) is a male individual around 39-43 years of age.

Early Metal Age sites in southeastern Tibet

Redilong (3260 masl)

The cemetery of Redilong is located near the Lancang River in Karuo. Five stone-cist burials were exposed on the surface, most of them were destroyed by water erosion. Only graves 3 and 5 contained well-preserved human bones, which were associated with a single bone needle and a bronze knife (*76*). In this study, we selected one individual from grave 3 to sample for calculus.

- 2002XCKM3 (DA798; cal. 926-418 BCE) is a 25-30-year-old individual.

Chugong (2243 masl)

Chugong is the lowest elevated site in our sample set, with an altitude of 2243 masl. Six stone-cist burials were excavated in 2019. Relatively few grave goods were found, including pottery, beads, and iron fragments. We sampled two individuals for this study.

- 2019 LYJQM1 (DA739; cal. 159 BCE- 26 CE, Beta-523131) is an elderly female, age cannot be determined.
- 2019 LYJQM6 (DA803; cal. 351-52 BCE, Beta-523135) is an adult female, age cannot be determined.

Agangrong (2800 masl)

Thirteen stone-cist burials at Agangrong were excavated during roadwork construction in 2016. Three burials were represented by complete skeletons, the remainder of the graves contained a paucity of human bones. Pottery, bronze weapons, iron fragments, and grinding stones accompanied these skeletons. We included two individuals from this group in this study.

- 2016 LBQAM5 (DA795; cal. 45 BCE- 75 CE) is a male individual, who died at an estimated age of 25 years.
- 2016 LBQAM13 (DA738) is a 39-43-year-old female.

Early Metal Age sites in western Qinghai

Pukar Gongma (4177 masl)

Pukar Gongma is the first stone-cist burial in western Qinghai to be excavated. The site is located on a modern high-altitude pasture next to the Nieqia River. Nine burials were excavated in 2013 (*77*). The subsistence economy at this site is unclear, owing to a lack of animal and plant remains. Only one sheep/goat skull was collected within these burials. Modern inhabitants near the site practice a mobile pastoral economy, likely also employed by ancient populations as the high-elevation environment presents significant challenges for farming. Remarkably, 5 of the 9 individuals at this burial site were subadults. We sampled three individuals in our research include:

- 2013QZPGM2 (DA796) is an individual with only a few grave goods, including one pottery jar, one stone axe, and a bronze knife.
- **2013QZPGM5 (DA797; cal. 1048-836 BCE, Beta-180102)** is a juvenile aged 10-11 years. This individual was interred with the most abundant artefacts of any grave in the group, including pottery, bronze and agate ornaments, seashells, and bone beads (fig. S10).
- **2013QZPGM6 (DA869; cal. 1014-836 BCE, Beta-583727)** is a 6-7-year-old child who has ambiguous sex characteristics. The only associated finding was a ceramic pot (fig. S10).

Text S3: Ancient dietary information in human dental calculus

Dental calculus, a white/light-brown mineralized biofilm developed from dental plaque (fig. S13), represents an excellent resource for studying the diet, health, and behavior of ancient populations (*78*–*80*), as well as the evolution of the oral microbiome (*81*–*83*). Dental plaque biofilm entraps diverse micro-remains from the mouth, including oral bacteria (>99%), host proteins, viruses, and food debris (*35*, *84*). Under specific oral environmental conditions (e.g., high calcium and phosphate ion levels, low pH) (*85*), accumulated dental plaque on the surface of the teeth becomes a mineralized matrix by building up layers during the life of the individual, thereby locking in abundant biomolecules. Modern dental calculus consists of around 80-85% inorganic crystals dominated by calcium phosphate, and about 15-20% organic remains composed primarily of oral bacteria, along with a more minor component of micro-remains, including proteins, phytoliths, starches, lipids, and DNA (*85*). Genomic and proteomic analysis of ancient human dental calculus suggests that the vast majority (~83-99%) of organic compounds are bacteria, followed by oral and immune proteins, with only a very minor component of proteins (~0.4%) and DNA (~0.01%) originating from dietary sources (*36*, *81*). Heavily calcified calculus has the potential to survive for thousands, even millions of years (*78*), making it an invaluable material for archaeological investigation.

Increasing research into ancient dental calculus over the past decade has greatly facilitated the reconstruction of human diets. While most studies of dental calculus focus on recovering plant dietary components through the analysis of phytoliths and starch remains (*86*), proteomic and genomic research has recently begun to enable higher resolution identification of dietary components absent or under-represented in microbotanic-based approaches. Several studies have successfully extracted dairy proteins in calculus, including the milk of sheep, goat, cattle, horse, and camel (*23*,*25*,*26*,*34*,*37*). Additionally, proteomics also has the potential to identify ancient plant proteins from dental calculus, such as cereals, legumes, oil plants, fruits, and spices (*40*,*87*).

The extent to which it is possible to reconstruct ancient diets from food remains in calculus is still not well understood for several reasons. Firstly, not all aspects of a person's diet are recorded in calculus; testing even modern samples reveals only a limited proportion of overall dietary breadth (*88*). Secondly, the formation rate of calculus exhibits significant individual variability, as a result of several factors, such as diet (with more protein and carbohydrate-rich diets supporting more calculus formation), chewing activity, salivary flow rate, genetic factors, and concentrations of calcium, phosphate, and silicon (*89*). Thirdly, the existence of notable biases in the adsorption and/or preservation of different food remains (*90*). Power and colleagues (*88*) report that phytoliths in chimpanzee calculus are a better reflection of long-lived dietary information than starch remains. Research indicates that milk proteins are more likely to be detected through palaeoproteomic methods than other food proteins, especially the whey protein β-lactoglobulin as a result of its notable resistance to enzymatic digestion, microbial proteolysis, and heat and acid environments (*34*,*38*,*91*,*92*). Therefore, dietary information that becomes encased in calculus is to some degree random, with concomitant challenges for dietary reconstruction, particularly with

regards to: 1) the full composition of an individual's diet during their lifetime; 2) the intensity of consumption of individual foods (*91,93*). It is important to note that while the presence of food proteins allows particular foods consumed to be identified, the absence of dietary proteins does not confirm that an individual did not consume a specific food. Despite these limitations, dental calculus does directly document the consumption of particular foodstuffs in specific individuals, sometimes with species-specific information about food sources and/or information about food sources that is difficult or impossible to acquire through other methods, and can thus be an extremely powerful tool for dietary studies in archaeology (*36*).

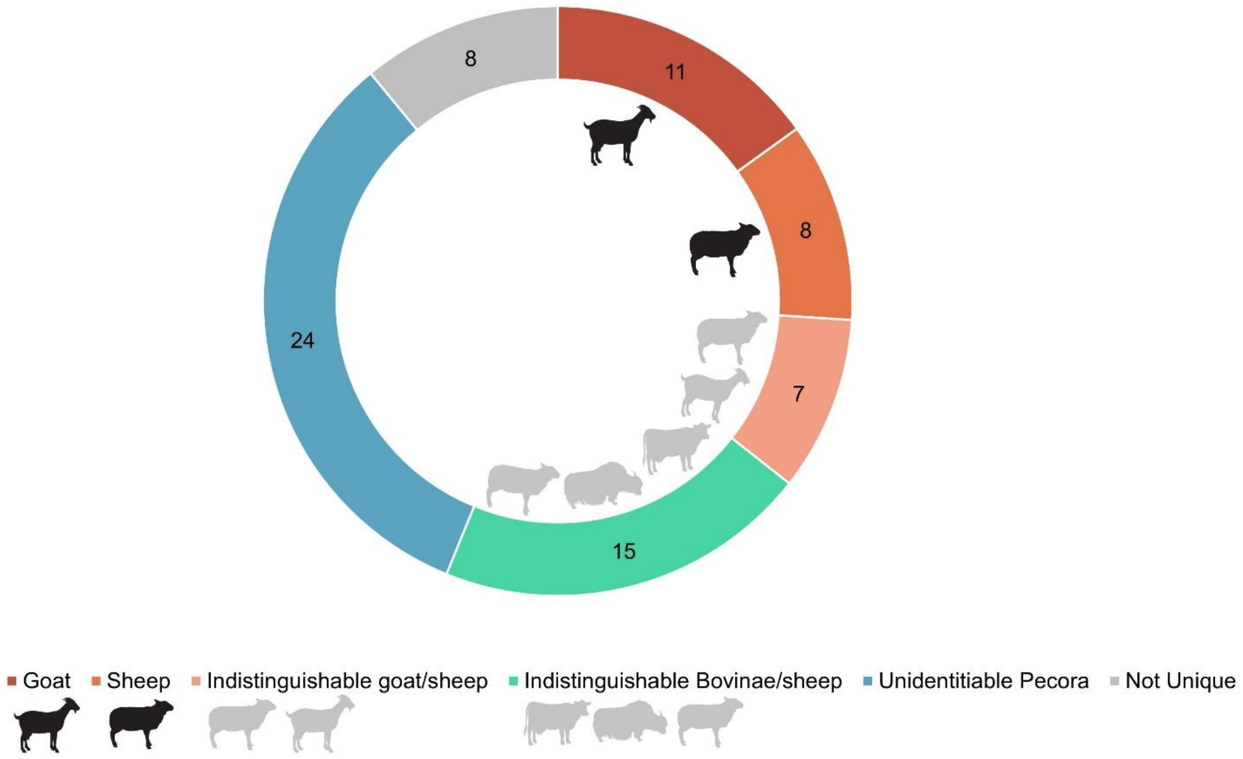

Fig. S1. Dairy livestock in the prehistoric Tibetan Plateau. Taxonomically assigned milk proteins (β-lactoglobulin) across all sites; the number represents the quantity of peptide spectral matches to each taxonomic group (confident attribution indicated by black icons, indistinguishable species attributions indicated in grey, details in Methods 'protein identification' section) (dataset S4).

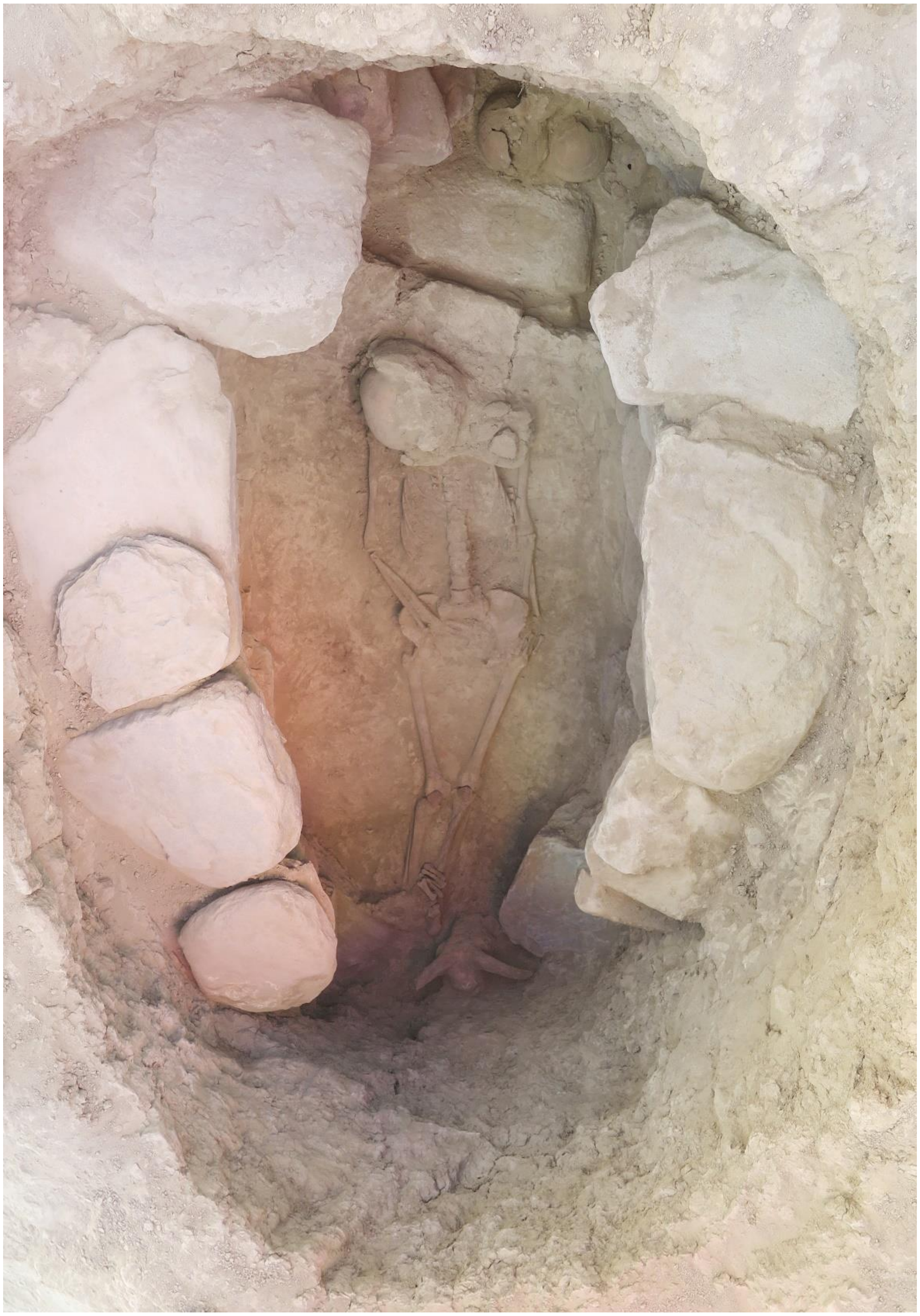

Fig. S2. The earliest studied skeleton from grave 2 (2017ZGM2) at Gepaseru. A goat skull was buried near her feet; cattle and goat bones were also recovered from this burial (Photograph: Shargan Wangdue).

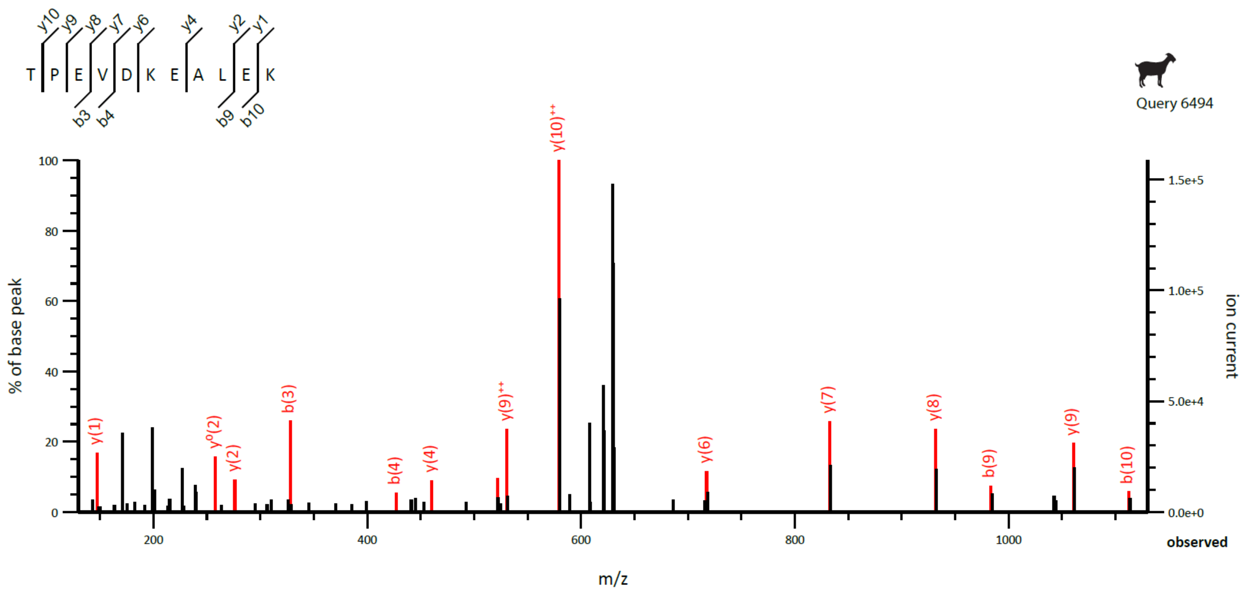

Fig. S3. MS/MS spectra showing *Capra* milk peptide from the dental calculus of the earliest dairying individual in this research at Gepaseru (DA793, cal. 1500-1312 BCE) in western Tibet.

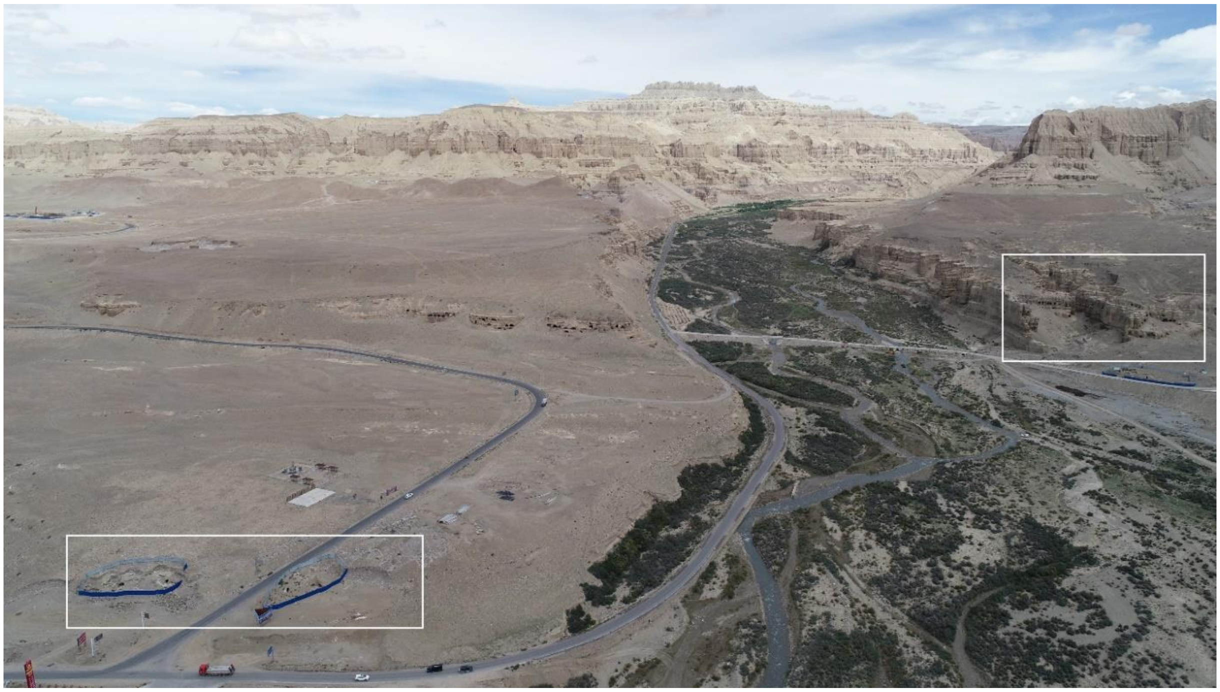

Fig. S4. Excavation areas in the Sangda Longo burial cluster. Excavation areas shown in the white squares (Photograph: Wei He).

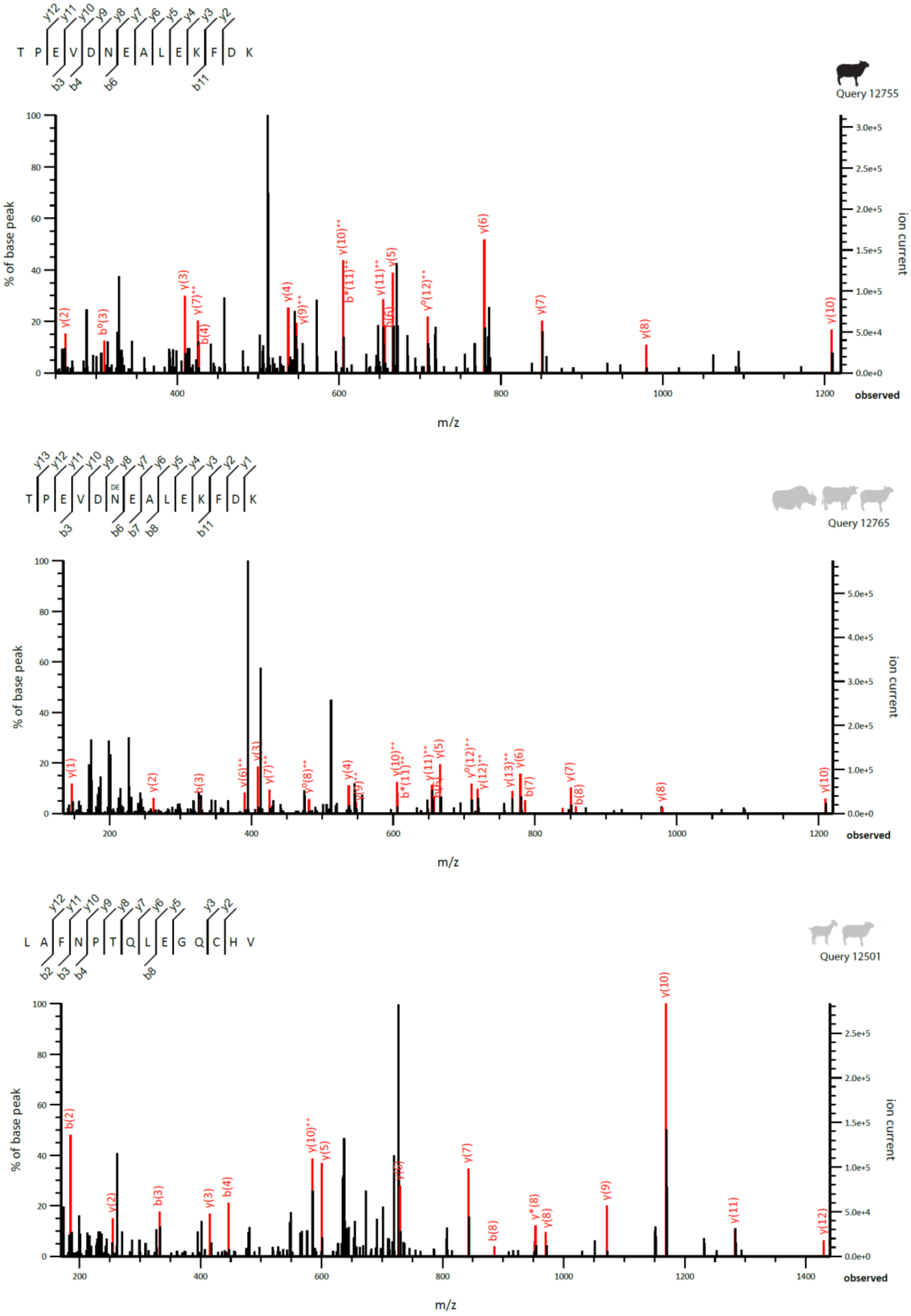

Fig. S5. MS/MS spectra showing whey protein BLG from the dental calculus of an elite burial at Sangda Longgo (DA741, cal. 120-306 CE) in western Tibet (Above: *Ovis*; middle: Bovinae or *Ovis*; below: Caprinae).

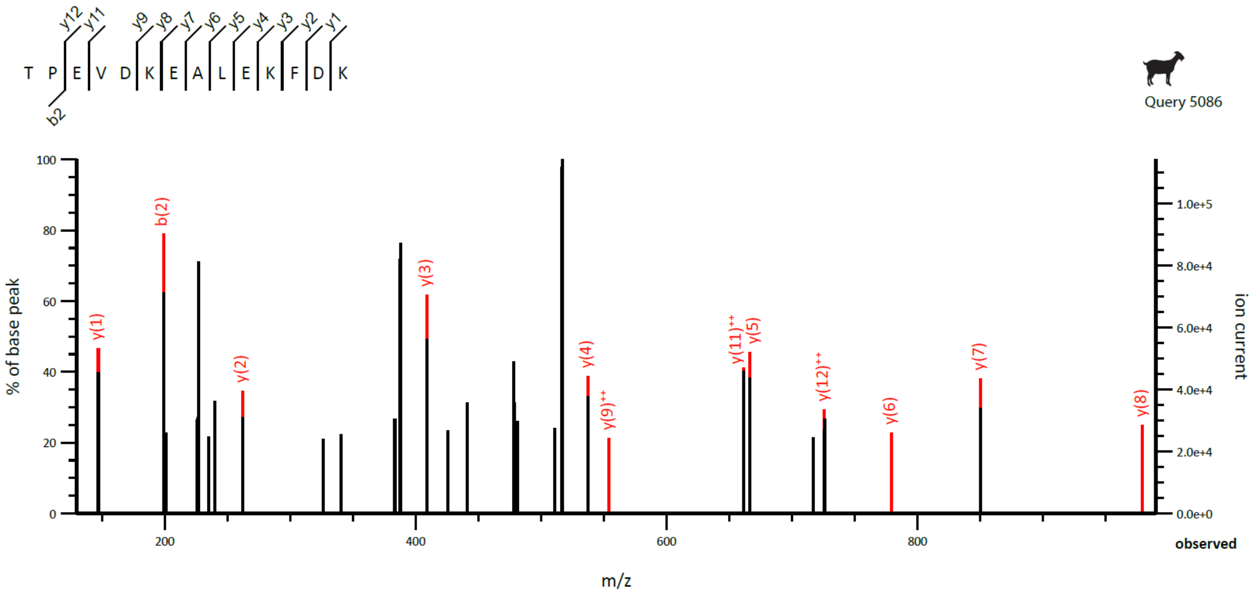

Fig. S6. MS/MS spectra showing *Capra* milk protein BLG from an individual at Sangda Longo (DA880, cal. 234-381 CE) in western Tibet.

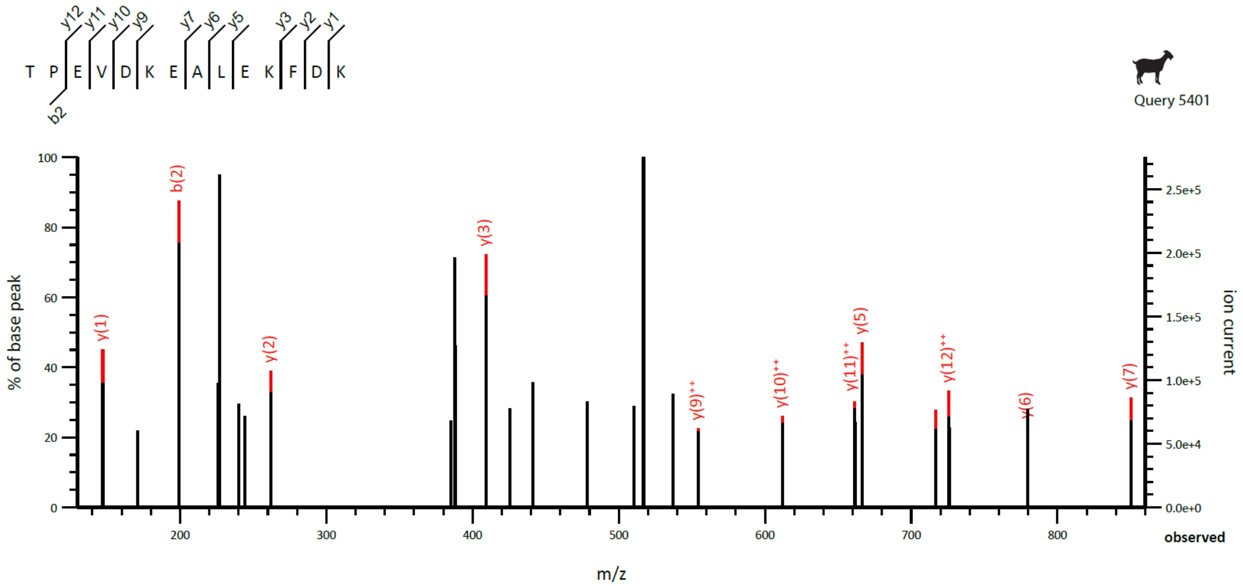

Fig S7. MS/MS spectra showing *Capra* BLG from an individual at Sangda Longo (DA881, cal. 196 BCE - 4 CE) in western Tibet.

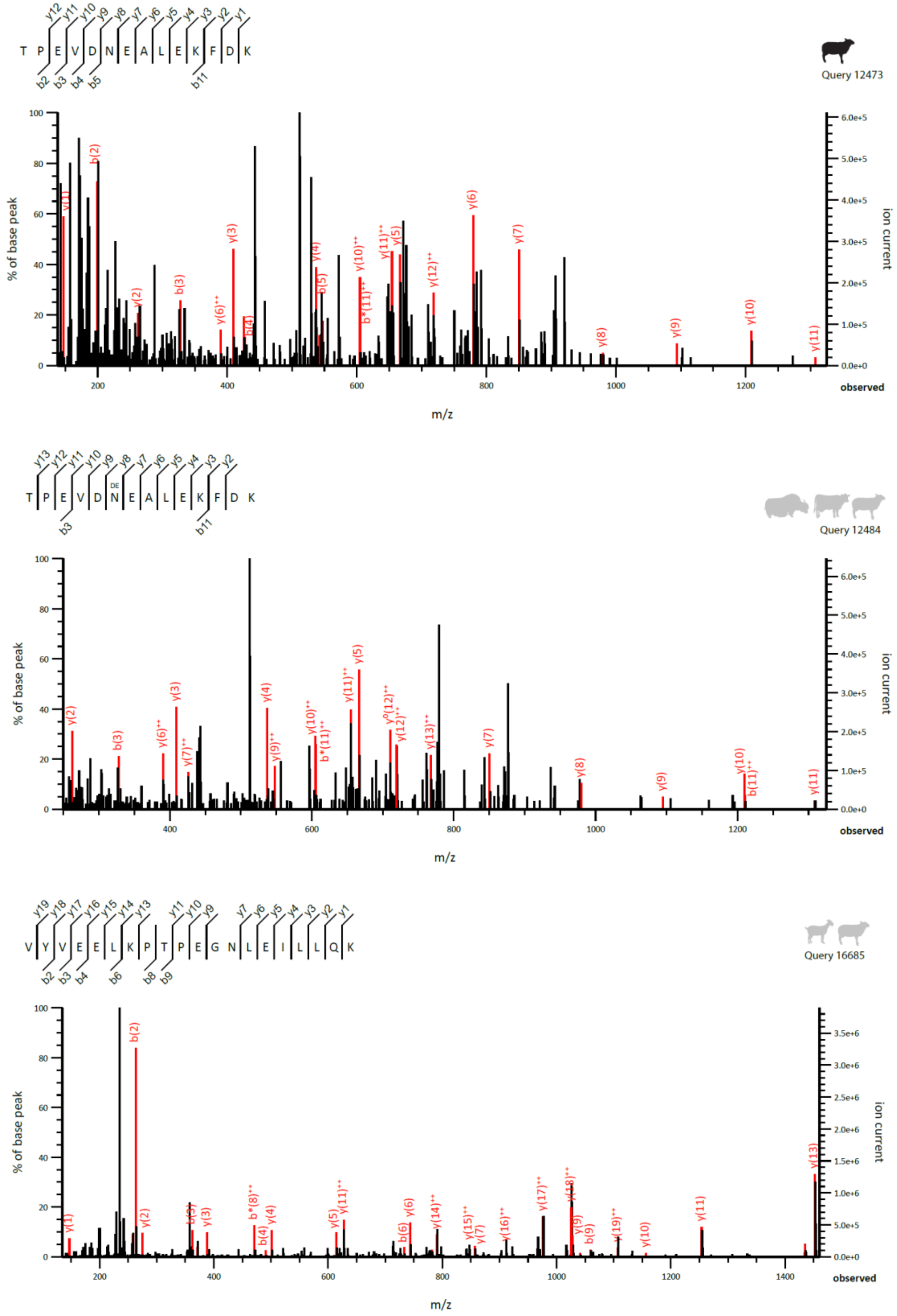

Fig. S8. MS/MS spectra showing whey protein β-lactoglobulin (BLG) from Malang (DA735, cal. 81-236 CE) in western Tibet. Species-specific information is indicated by animal icons (Above: *Ovis*; middle: Bovinae or *Ovis*; below: Caprinae).

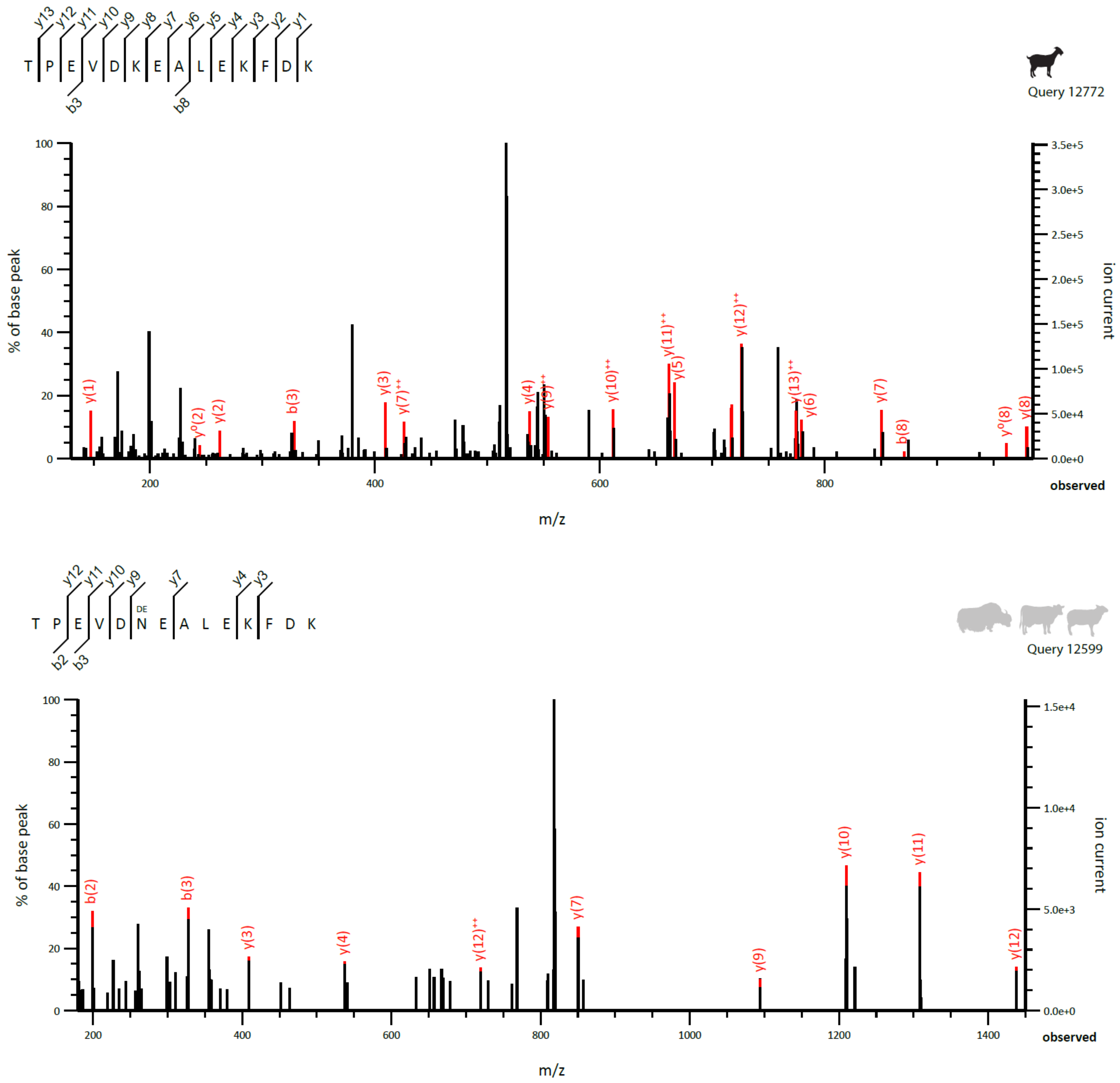

Fig. S9. MS/MS spectra showing whey protein BLG from Khunglung Satra (DA808, cal. 250-411 CE) in western Tibet (Above: *Capra*; below: Bovinae or *Ovis*).

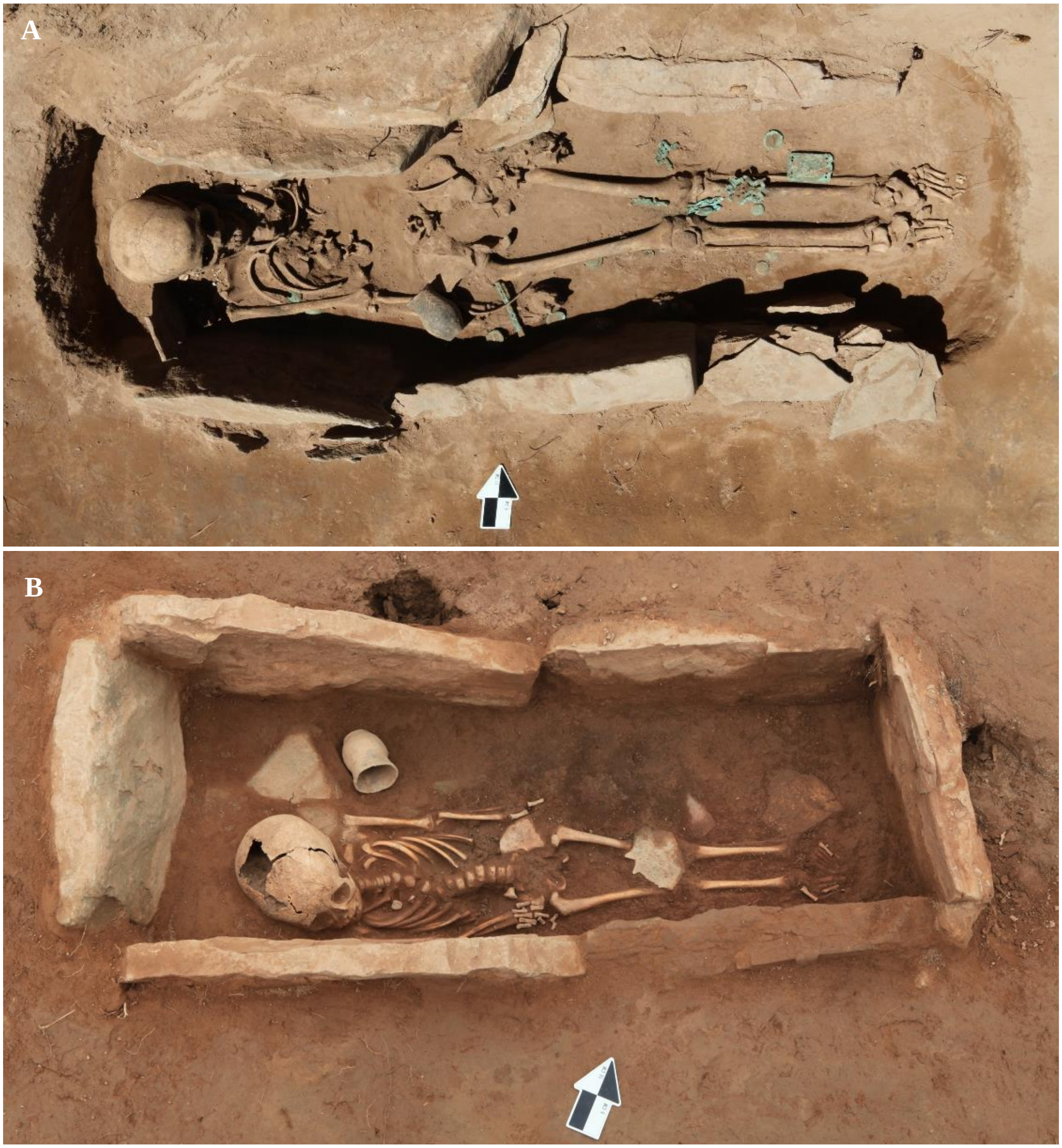

Fig. S10. Human skeletons from Pukar Gongma on the northern Tibetan Plateau. A, A juvenile aged 10-11 years in grave 5, accompanied by the most abundant grave goods of any burial in this burial cluster. **B**, A 6-7-year-old child from grave 6, buried with only one ceramic vessel (Photograph: Yuanhong He).

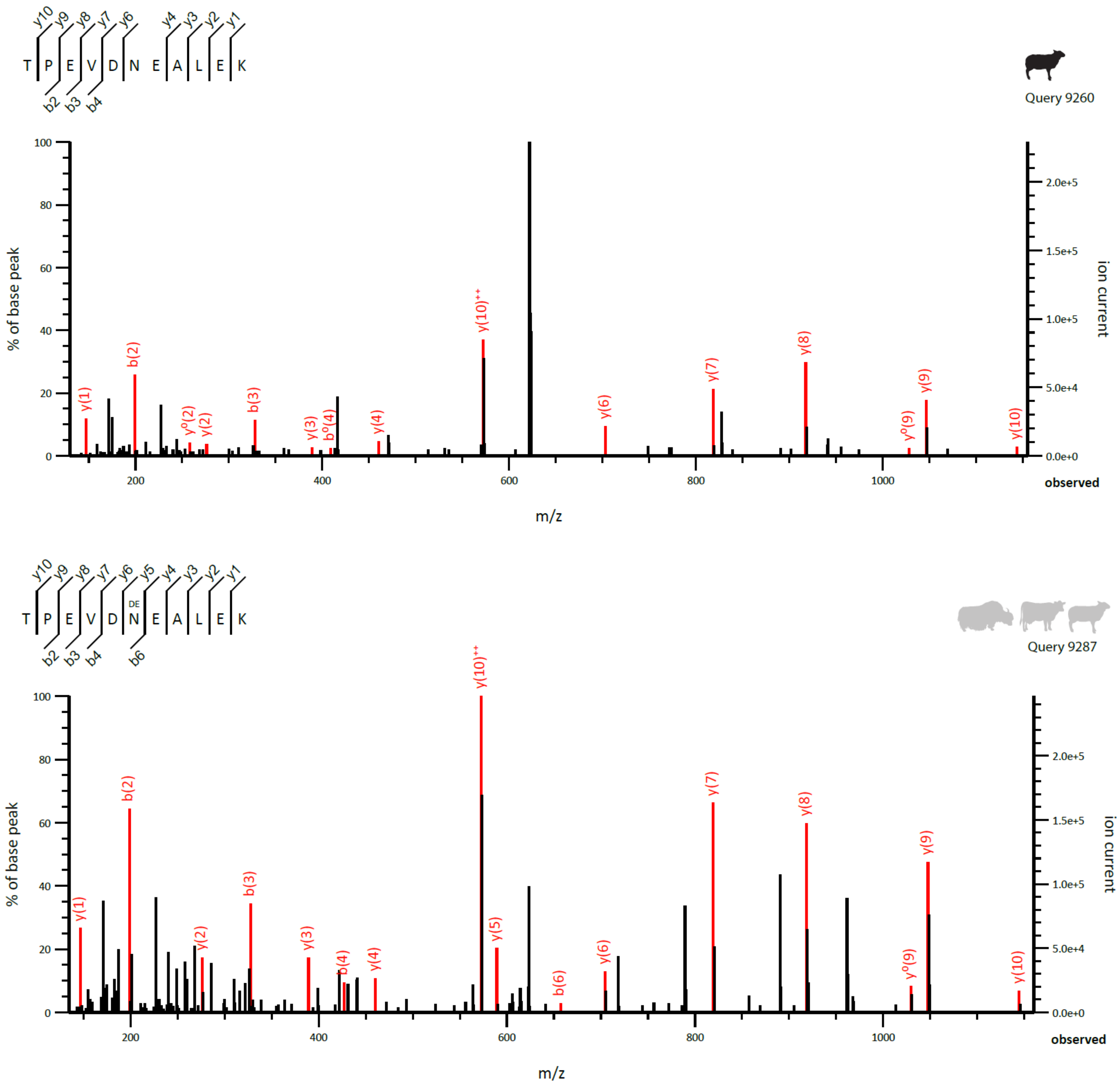

Fig. S11. MS/MS spectra showing milk protein BLG from an individual at Pukar Gongma (DA797, cal. 1048-836 BCE) in western Qinghai (Above: *Ovis*; below: Bovinae or *Ovis*).

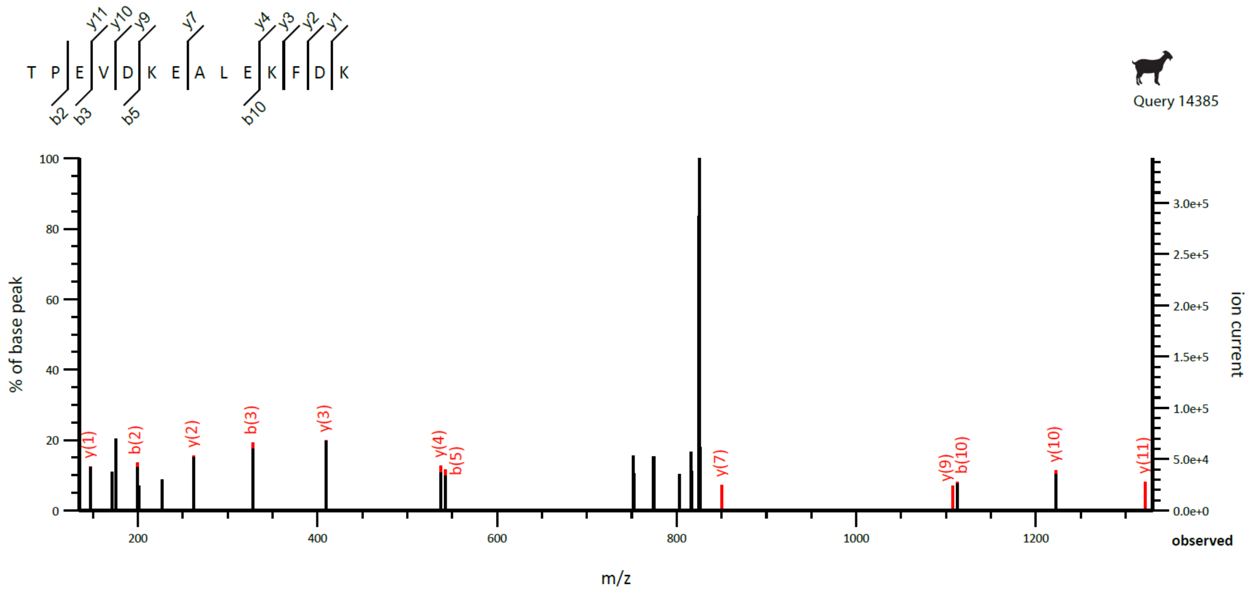

Fig. S12. MS/MS spectra showing *Capra* BLG from Pukar Gongma (DA869, cal. 1014-836 BCE) in western Qinghai.

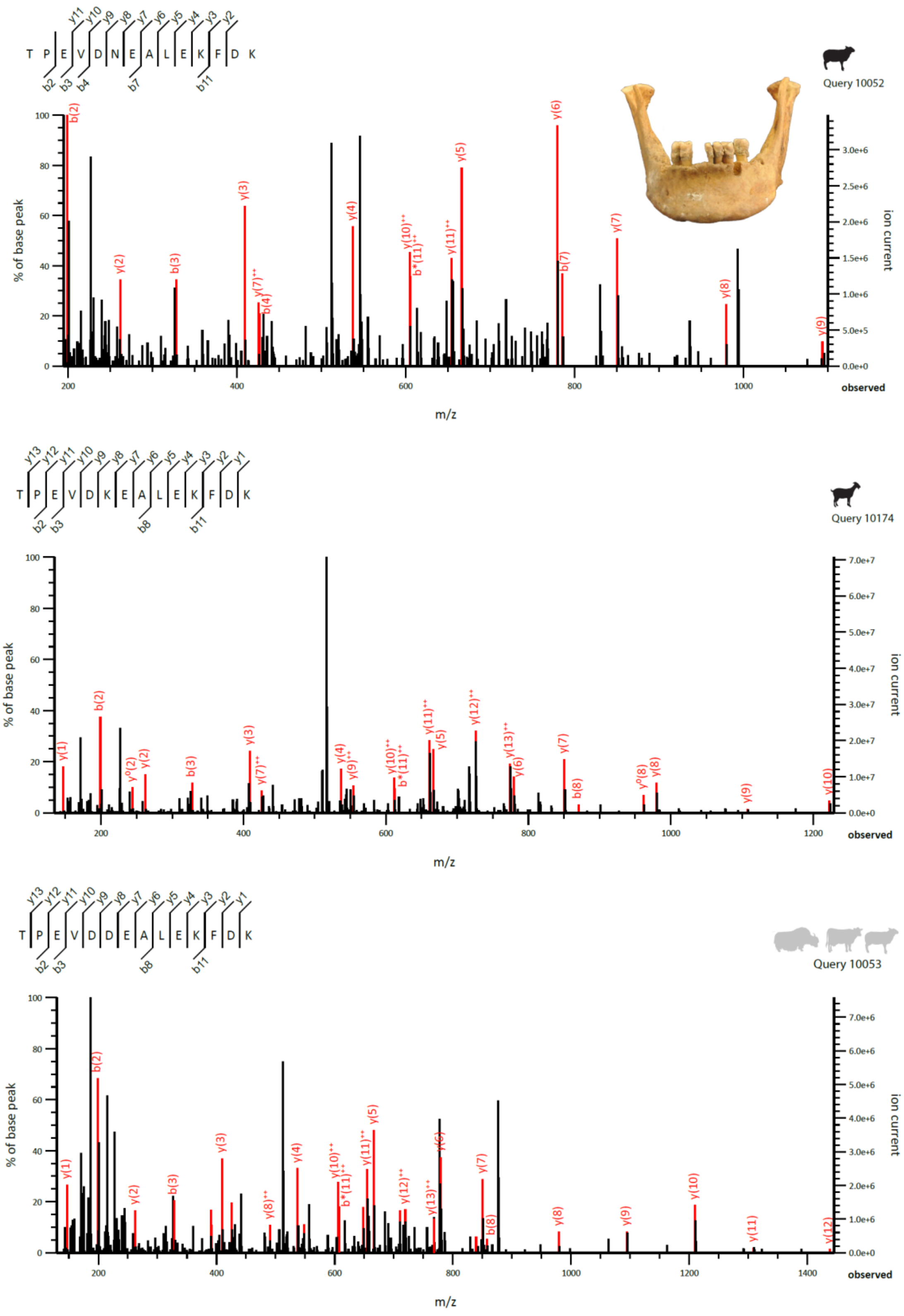

Fig. S13. MS/MS spectra showing milk protein BLG from an individual at Ounie (DA742, cal. 601-758 CE) in northern Tibet (Above: *Ovis*; middle: *Capra*; below: Bovinae or *Ovis*).

Table S1. The distribution of total land, cropland, and grassland in different elevation ranges of the Tibetan Plateau (*65,66,68,69*).

Elevation (masl)	Total Area (km^2)	Area (%)	Cropland (km^2)	Grassland (km^2)	Cropland/ total area (%)	Grassland/ total area (%)	Relative proportion of cropland (%)	Relative proportion of grassland (%)
<2500	31,630	1.24%	3268	9856	10.3%	31.2%	24.9%	75.1%
2500-3500	356,651	14.03%	9364	145,154	2.6%	40.7%	6.1%	93.9%
>3500	2,154,019	84.73%	5071	1,584,303	0.2%	73.6%	0.3%	99.7%

Table S2. **Summary of contextual information and dairy peptide identifications for the individuals examined in the present study.**

Region	Site name	Site code	Lab code	Elevation	Milk protein taxa identified
Western Tibet	Gepaseru	2017ZGM2	DA793	3943	*Capra*
		2019ZGM6	DA736		No milk identified
		2017ZGM10	DA800		Fail to pass preservation threshold
		2019ZGM7	DA871		Fail to pass preservation threshold
		2018ZGM1	DA872		No milk identified
		2019ZGM4	DA873		Fail to pass preservation threshold
	Khunglung Satra	2019QLSZM1	DA808	4282	*Capra,* Bovinae or *Ovis*
	Gelintang	1999ZPGM6	DA799	4200	Fail to pass preservation threshold
		1999ZPGM7	DA870		Fail to pass preservation threshold
	Sangda Longo	2017ZSM6	DA804	3700	No milk identified
		2017ZSM7	DA885		Fail to pass preservation threshold
		2018ZSEM3	DA880		*Capra*
		2018ZSWM3	DA881		*Capra*
		2018ZSM5	DA882		No milk identified
		2019ZSEM25	DA883		No milk identified
		2019ZSEM28	DA741		*Ovis*, Bovinae or *Ovis*, Caprinae
		2019ZSEM31	DA884		Fail to pass preservation threshold
		2019ZSEM26:129	DA805		No milk identified
		2019ZSEM26:179	DA886		Fail to pass preservation threshold
		2019ZSEM26:182	DA806		No milk identified
		2019ZSEM26:184	DA887		Fail to pass preservation threshold
		2019ZSEM26:211	DA888		No milk identified
	Jiweng	2018ZPJM7	DA877	4085	No milk identified
	Malang	2019ZMM1	DA735	3910	*Ovis,* Bovinae or *Ovis*, Caprinae
Northern Tibet	Zhajiongema	2019BZM2	DA807	4648	Fail to pass preservation threshold
	Ounie	2018BOM5	DA742	4654	*Capra, Ovis,* Bovinae or *Ovis*
Western Qinghai	Pukar Gongma	2013QZPGM2	DA796	4177	No milk identified
		2013QZPGM5	DA797		*Ovis*, Bovinae or *Ovis*
		2013QZPGM6	DA869		*Capra*
Eastern Tibet	Agangrong	2016LBQAM5	DA795	2800	Fail to pass preservation threshold
		2016LBQAM13	DA738		No milk identified
	Redilong	2002XCKM3	DA798	3260	Fail to pass preservation threshold
	Chugong	2019LYJQM1	DA739	2243	No milk identified
		2019LYJQM6	DA803		No milk identified
Central Tibet	Thing	2017LZTCM1-60	DA794	3314	Fail to pass preservation threshold
		2017LZTCM1-64	DA740		Fail to pass preservation threshold
	Nubda	2019RRNCM1	DA737	3833	No milk identified
		2019RRNCM4	DA875		No milk identified
		2019RRNCG	DA874		No milk identified
	Neu Lacok	2003LWZLJM6	DA889	3700	No milk identified

Table S3: Information of cropland area and archaeological sites in arable and non-arable regions.

City/ Prefecture	Total area (km^2)	Cropland area* (km^2)	Cropland/ Total land (%)	Cropland soil quality†	Arable/ Non-arable§	Archaeological sites
Ngari	337,175	30	0.01%	9.9	Non-arable	Gepaseru, Jiweng, Khunglung Satra, Malang, Sangda Longo
Nagqu	352,192	79	0.02%	9.7	Non-arable	Ounie, Zhajiongema
Yushu	210,300	134	0.06%	#	Non-arable	Pukar Gongma
Nyingchi	114,215	264	0.23%	6.0	Arable	Agangrong, Chugong
Qamdo	109,817	717	0.65%	8.8	Arable	Redilong
Xigaze	179,903	1357	0.75%	8.9	Arable	Nubda
Shannan	79,254	646	0.81%	8.2	Arable	Thing
Lhasa	29,634	555	1.87%	8.9	Arable	Neu Lacok

* We collected data from *Mountain Geoecology and Sustainable Development of the Tibetan Plateau* (*68*).

† The soil quality of cropland is divided into 10 ranks (1-10) based on multiproxy investigations by the department of agriculture and rural affairs of Tibet Autonomous Region in 2019, the higher score means poorer soil quality for cultivation. (http://xzgbnc.org/show-121317.html).

§ The percentage of cropland area/total land area ⩾ 0.2% was separated into arable group.

# Lack of investigated data from this region.

Table S4. Information on chronology, elevation, and age for individuals whose calculus yielded dairy proteins.

Proteomics lab No.	Archaeological ID (Site name)	Elevation (masl)	^{14}C lab code	Calibrated ^{14}C (BCE/CE)*	Conventional ^{14}C age (BP)	Osteological age†	Peptide spectral matches (PSMs)	Species Identified‡
DA793	2017ZGM2 (Gerpaseru)	3943	GU55771	1500-1312 BCE	3149+/- 30	25-35	4	Goat
DA797	2013QZPGM5 (Pukar Gongma)	4177	Beta-180102	1048-836 BCE§	2800+/-35	10-11	7	Sheep, indistinguishable sheep/cattle/yak
DA869	2013QZPGM6 (Pukar Gongma)	4177	Beta-583727	1014-836 BCE	2790+/-30	6-7	3	Goat
DA881	2018ZSWM3 (Sangda Longo)	3700	Beta-513146	196 BCE- 4 CE	2090+/- 30	40-50	3	Goat
DA735	2019ZMM1 (Malang)	3910	Beta-547974	81-236 CE	1880+/- 30	~25	6	Sheep, indistinguishable sheep/cattle/yak, indistinguishable sheep/goat
DA741	2019ZSEM28 (Sangda Longo)	3700	Beta-543653	120-306 CE	1850+/- 30	40-55	16	Sheep, indistinguishable sheep/cattle/yak, indistinguishable sheep/goat
DA880	2018ZSEM3 (Sangda Longo)	3700	Beta-513147	234-381 CE	1760+/- 30	25-35	3	Goat
DA808	2017QLSZM1 (Khunglung Satra)	4282	Beta-575047	250-411 CE	1720+/-30	25-35	5	Goat, indistinguishable sheep/cattle/yak
DA742	2018BOM5 (Ounie)	4654	Beta-513157	601-758 CE	1380 +/- 30	40-55	26	Goat, sheep, indistinguishable sheep/cattle/yak

* Date range reported as a 95.4% confidence interval, for more details see Methods 'Radiocarbon dating' section.

† Age estimation based on the morphological characteristics of human skeletons and teeth.

‡ Dairy protein taxonomic assignment based on amino acid sequence (Methods 'protein identification' section).

§ All the radiocarbon dating results are newly presented with the exception of DA797 (*6*).

Supplementary Datasets S1-4. (separate file)

Dataset S1. Previously published domesticated animal bone evidence from archaeological sites on the Tibetan Plateau

Dataset S2. OSSD assessment data for each studied individual

Dataset S3. Protein information of studied individuals and lab controls (search under OSSD)

Dataset S4. Peptide sequence information for Figure 3

REFERENCES AND NOTES

1. J. Qiu, China: The third pole. *Nature* **454**, 393–396 (2008).

2. Institute of Medicine, *Nutritional Needs in Cold and High-Altitude Environments: Applications for Military Personnel in Field Operations* (The National Academy Press, 1996).

3. N. Petousi, P. A. Robbins, Human adaptation to the hypoxia of high altitude: The Tibetan paradigm from the pregenomic to the postgenomic era. *J. Appl. Physiol.* **116**, 875–884 (2014).

4. X. Yi, Y. Liang, E. Huerta-Sanchez, X. Jin, Z. X. P. Cuo, J. E. Pool, N. Xu, H. Jiang, N. Vinckenbosch, T. S. Korneliussen, H. C. Zheng, T. Liu, W. M. He, K. Li, R. B. Luo, X. F. Nie, H. L. Wu, M. R. Zhao, H. Z. Cao, J. Zou, Y. Shan, S. Z. Li, Q. Yang, Asan, P. X. Ni, G. Tian, J. M. Xu, X. Liu, T. Jiang, R. H. Wu, G. Y. Zhou, M. F. Tang, J. J. Qin, T. Wang, S. J. Feng, G. H. Li, Huasang, J. Luosang, W. Wang, F. Chen, Y. D. Wang, X. G. Zheng, Z. Li, Z. Bianba, G. Yang, X. P. Wang, S. H. Tang, G. Y. Gao, Y. Chen, Z. Luo, L. Gusang, Z. Cao, Q. H. Zhang, W. H. Ouyang, X. L. Ren, H. Q. Liang, H. S. Zheng, Y. B. Huang, J. X. Li, L. Bolund, K. Kristiansen, Y. R. Li, Y. Zhang, X. Q. Zhang, R. Q. Li, S. G. Li, H. M. Yang, R. Nielsen, J. Wang, J. Wang, Sequencing of 50 human exomes reveals adaptation to high altitude. *Science* **329**, 75–78 (2010).

5. J. Yang, Z.-B. Jin, J. Chen, X.-F. Huang, X.-M. Li, Y.-B. Liang, J.-Y. Mao, X. Chen, Z. Zheng, A. Bakshi, D.-D. Zheng, M.-Q. Zheng, N. R. Wray, P. M. Visscher, F. Lu, J. Qu, Genetic signatures of high-altitude adaptation in Tibetans. *Proc. Natl. Acad. Sci. U.S.A.* **114**, 4189–4194 (2017).

6. M. Ding, T. Wang, A. M.-S. Ko, H. Chen, H. Wang, G. Dong, H. Lu, W. He, S. Wangdue, H. Yuan, Y. He, L. Cai, Z. Chen, G. Hou, D. Zhang, Z. Zhang, P. Cao, Q. Dai, X. Feng, M. Zhang, H. Wang, M. A. Yang, Q. Fu, Ancient mitogenomes show plateau populations from last 5200 years partially contributed to present-day Tibetans. *Proc. Biol. Sci.* **287**, 20192968 (2020).

7. C. Jeong, G. Alkorta-Aranburu, B. Basnyat, M. Neupane, D. B. Witonsky, J. K. Pritchard, C. M. Beall, A. Di Rienzo, Admixture facilitates genetic adaptations to high altitude in Tibet. *Nat. Commun.* **5**, 3281 (2014).

8. C.-C. Liu, D. Witonsky, A. Gosling, J. H. Lee, H. Ringbauer, R. Hagan, N. Patel, R. Stahl, J. Novembre, M. Aldenderfer, C. Warinner, A. Di Rienzo, C. Jeong, Ancient genomes from the Himalayas illuminate the genetic history of Tibetans and their Tibeto-Burman speaking neighbors. *Nat. Commun.* **13**, 1203 (2022).

9. F. H. Chen, G. H. Dong, D. J. Zhang, X. Y. Liu, X. Jia, C. B. An, M. M. Ma, Y. W. Xie, L. Barton, X. Y. Ren, Z. J. Zhao, X. H. Wu, M. K. Jones, Agriculture facilitated permanent human occupation of the Tibetan Plateau after 3600 B.P. *Science* **347**, 248–250 (2015).

10. D. X. Fu, R. W. Ruan, X. M. Dai, Y. M. Liu, A study on ancient barley, wheat and millet discovered at Changguogou of Tibet. *Acta Agron. Sin.* **26**, 392–398 (2000).

11. J. d’Alpoim Guedes, H. Lu, Y. Li, R. N. Spengler, X. Wu, M. S. Aldenderfer, Moving agriculture onto the Tibetan plateau: The archaeobotanical evidence. *Archaeol. Anthropol. Sci.* **6**, 255–269 (2014).

12. Y. Gao, J. S. Yang, Z. K. Ma, Y. Tong, X. Y. Yang, New evidence from the Qugong site in the central Tibetan Plateau for the prehistoric Highland Silk Road. *Holocene* **31**, 230–239 (2021).

13. L. Tang, H. Lu, J. Song, S. Wangdue, X. Chen, Z. Zhang, X. Liu, N. Boivin, R. N. Spengler III, The transition to a barley-dominant cultivation system in Tibet: First millennium BC archaeobotanical evidence from Bangga. *J. Anthropol. Archaeol.* **61**, 101242 (2021).

14. K.-H. Knörzer, 3000 years of agriculture in a valley of the High Himalayas. *Veg. Hist. Archaeobot.* **9**, 219–222 (2000).

15. N. Tashi, Y. H. Liu, and T. Partap, *Making Tibet Food Secure: Assessment of Scenarios* (International Centre for Integrated Mountain Development, 2002).

16. G. Miehe, S. Miehe, K. Kaiser, C. Reudenbach, L. Behrendes, L. Duo, F. Schlütz, L. Duo, F. Schlütz, How old is pastoralism in Tibet? An ecological approach to the making of a Tibetan landscape. *Palaeogeogr. Palaeoclimatol. Palaeoecol.* **276**, 130–147 (2009).

17. D.-D. Wu, C.-P. Yang, M.-S. Wang, K.-Z. Dong, D.-W. Yan, Z.-Q. Hao, S.-Q. Fan, S.-Z. Chu, Q.-S. Shen, L.-P. Jiang, Y. Li, L. Zeng, H.-Q. Liu, H.-B. Xie, Y.-F. Ma, X.-Y. Kong, S.-L. Yang, X.-X. Dong, A. Esmailizadeh, D. M. Irwin, X. Xiao, M. Li, Y. Dong, W. Wang, P. Shi, H.-P. Li, Y.-H. Ma, X. Gou, Y.-B. Chen, Y.-P. Zhang, Convergent genomic signatures of high-altitude adaptation among domestic mammals. *Natl. Sci. Rev.* **7**, 952–963 (2020).

18. M. C. Goldstein, C.M. Beall, *Nomads of Western Tibet: The Survival of a Way of Life*. (Waveland Press, 1990).

19. R. B. Ekvall, *Fields on the Hoof: Nexus of Tibetan Nomadic Pastoralism* (Odyssey, 1968).

20. D. J. Miller, Tough times for Tibetan nomads in western China: Snowstorms, settling down, fences and the demise of traditional nomadic pastoralism. *Nomad. People.* **4**, 83–109 (2000).

21. R. P. Evershed, S. Payne, A. G. Sherratt, M. S. Copley, J. Coolidge, D. Urem-Kotsu, K. Kotsakis, M. Özdoğan, A. E. Özdoğan, O. Nieuwenhuyse, P. M. M. G. Akkermans, D. Bailey, R.-R. Andeescu, S. Campbell, S. Farid, I. Hodder, N. Yalman, M. Özbaşaran, E. Biçakci, Y. Garfinkel, T. Levy, M. M. Burton, Earliest date for milk use in the Near East and southeastern Europe linked to cattle herding. *Nature* **455**, 528–531 (2008).

22. O. E. Craig, J. Chapman, C. Heron, L. H. Willis, L. Bartosiewicz, G. Taylor, A. Whittle, M. Collins, Did the first farmers of central and eastern Europe produce dairy foods? *Antiquity* **79**, 882–894 (2005).

23. M. Bleasdale, K. K. Richter, A. Janzen, S. Brown, A. Scott, J. Zech, S. Wilkin, K. Wang, S. Schiffels, J. Desideri, M. Besse, J. Reinold, M. Saad, H. Babiker, R. C. Power, E. Ndiema, C. Ogola, F. K. Manthi, M. Zahir, M. Petraglia, C. Trachsel, P. Nanni, J. Grossmann, J. Hendy,

A. Crowther, P. Roberts, S. T. Goldstein, N. Boivin, Ancient proteins provide evidence of dairy consumption in eastern Africa. *Nat. Commun.* **12**, 632 (2021).

24. A. R. Ventresca Miller, S. Wilkin, J. Hendy, T. Turbat, D. Batsukh, N. Bayarkhuu, P.-H. Giscard, J. Bemmann, J. Bayarsaikhan, B. K. Miller, J. Clark, P. Roberts, N. Boivin, The spread of herds and horses into the Altai: How livestock and dairying drove social complexity in Mongolia. *PLOS ONE* **17**, e0265775 (2022).

25. C. Jeong, S. Wilkin, T. Amgalantugs, A. S. Bouwman, W. T. T. Taylor, R. W. Hagan, S. Bromage, S. Tsolmon, C. Trachsel, J. Grossmann, J. Littleton, C. A. Makarewicz, J. Krigbaum, M. Burri, A. Scott, G. Davaasambuu, J. Wright, F. Irmer, E. Myagmar, N. Boivin, M. Robbeets, F. J. Rühli, J. Krause, B. Frohlich, J. Hendy, C. Warinner, Bronze Age population dynamics and the rise of dairy pastoralism on the eastern Eurasian steppe. *Proc. Natl. Acad. Sci. U.S.A.* **115**, E11248–E11255 (2018).

26. S. Wilkin, A. Ventresca Miller, W. T. T. Taylor, B. K. Miller, R. W. Hagan, M. Bleasdale, A. Scott, S. Gankhuyg, A. Ramsøe, S. Uliziibayar, C. Trachsel, P. Nanni, J. Grossmann, L. Orlando, M. Horton, P. W. Stockhammer, E. Myagmar, N. Boivin, C. Warinner, J. Hendy, Dairy pastoralism sustained eastern Eurasian steppe populations for 5,000 years. *Nat. Ecol. Evol.* **4**, 346–355 (2020).

27. F. Zhang, C. Ning, A. Scott, Q. M. Fu, R. Bjørn, W. Y. Li, D. Wei, W. J. Wang, L. Y. Fan, I. Abuduresule, X. J. Hu, Q. R. Ruan, A. Niyazi, G. H. Dong, P. Cao, F. Liu, Q. Y. Dai, X. T. Feng, R. W. Yang, Z. H. Tang, P. C. Ma, C. X. Li, S. Z. Gao, Y. Xu, S. H. Wu, S. Q. Wen, H. Zhu, H. Zhou, M. Robbeets, V. Kumar, J. Krause, C. Warinner, C. Jeong, Y. Q. Cui, The genomic origins of the Bronze Age Tarim Basin mummies. *Nature* **599**, 256–261 (2021).

28. M. Xie, A. Shevchenko, B. Wang, A. Shevchenko, C. Wang, Y. Yang, Identification of a dairy product in the grass woven basket from Gumugou Cemetery (3800 BP, northwestern China). *Quat. Int.* **426**, 158–165 (2016).

29. Y. Yang, A. Shevchenko, A. Knaust, I. Abuduresule, W. Li, X. Hu, C. Wang, A. Shevchenko, Proteomics evidence for kefir dairy in Early Bronze Age China. *J. Archaeol. Sci.* **45**, 178–186 (2014).

30. S. Payne, Kill-off patterns in sheep and goats: The Mandibles from Aşvan Kale. *Anatol. Stud.* **23**, 281–303 (1973).

31. Y. S. Zamarayeva, A. V. Kistova, N. N. Pimenova, K. V. Reznikova, N. N. Seredkina, Taymyr reindeer herding as a branch of the economy and a fundamental social identification practice for indigenous peoples of the Siberian Arctic. *Mediterr. J. Soc. Sci.* **6**, 225–232 (2015).

32. G. Wang, L. Hua, V. R. Squires, G. Du, What road should the grazing industry take on pastoral land in China? *AIMS Agric. Food* **2**, 354–369 (2017).

33. A. Scott, S. Reinhold, T. Hermes, A. A. Kalmykov, A. Belinskiy, A. Buzhilova, N. Berezina, A. R. Kantorovich, V. E. Maslov, F. Guliyev, B. Lyonnet, P. Gasimov, B. Jalilov, J. Eminli, E. Iskandarov, E. Hammer, S. E. Nugent, R. Hagan, S. V. Sharapova, R. Krause, M. Karapetian, E. Stolarczyk, J. Krause, S. Hansen, W. Haak, C. Warinner, Emergence and intensification of dairying in the Caucasus and Eurasian steppes. *Nat. Ecol. Evol.* **6**, 813–822 (2022).

34. C. Warinner, J. Hendy, C. Speller, E. Cappellini, R. Fischer, C. Trachsel, J. Arneborg, N. Lynnerup, O. E. Craig, D. M. Swallow, A. Fotakis, R. J. Christensen, J. V. Olsen, A. Liebert, N. Montalva, S. Fiddyment, S. Charlton, M. Mackie, A. Canci, A. Bouwman, F. Rühli, M. T. P. Gilbert, M. J. Collins, Direct evidence of milk consumption from ancient human dental calculus. *Sci. Rep.* **4**, 7104 (2014).

35. J. L. Metcalf, L. K. Ursell, R. Knight, Ancient human oral plaque preserves a wealth of biological data. *Nat. Genet.* **46**, 321–323 (2014).

36. J. Hendy, C. Warinner, A. Bouwman, M. J. Collins, S. Fiddyment, R. Fischer, R. Hagan, C. A. Hofman, M. Holst, E. Chaves, L. Klaus, G. Larson, M. Mackie, K. McGrath, A. Z.

Mundorff, A. Radini, H. Y. Rao, C. Trachsel, I. M. Velsko, C. F. Speller, Proteomic evidence of dietary sources in ancient dental calculus. *Proc. R. Soc. B Biol. Sci.* **285**, 20180977 (2018).

37. S. Charlton, A. Ramsøe, M. Collins, O. E. Craig, R. Fischer, M. Alexander, C. F. Speller, New insights into Neolithic milk consumption through proteomic analysis of dental calculus. *Archaeol. Anthropol. Sci.* **11**, 6183–6196 (2019).

38. A. Ramsøe, M. Crispin, M. Mackie, K. McGrath, R. Fischer, B. Demarchi, M. J. Collins, J. Hendy, C. Speller, Assessing the degradation of ancient milk proteins through site-specific deamidation patterns. *Sci. Rep.* **11**, 7795 (2021).

39. S. Wilkin, A. Ventresca Miller, R. Fernandes, R. Spengler, W. T.-T. Taylor, D. R. Brown, D. Reich, D. J. Kennett, B. J. Culleton, L. Kunz, C. Fortes, A. Kitova, P. Kuznetsov, A. Epimakhov, V. F. Zaibert, A. K. Outram, E. Kitov, A. Khokhlov, D. Anthony, N. Boivin, Dairying enabled early bronze age Yamnaya steppe expansions. *Nature* **598**, 629–633 (2021).

40. J. Hendy, A. C. Colonese, I. Franz, R. Fernandes, R. Fischer, D. Orton, A. Lucquin, L. Spindler, J. Anvari, E. Stroud, P. F. Biehl, C. Speller, N. Boivin, M. Mackie, R. R. Jersie-Christensen, J. V. Olsen, M. J. Collins, O. E. Craig, E. Rosenstock, Ancient proteins from ceramic vessels at Çatalhöyük West reveal the hidden cuisine of early farmers. *Nat. Commun.* **9**, 4064 (2018).

41. T. Tashi, S. Wangdue, L. Xi, The exaction report of the Gepaseru burial in 2017, Zanda County, Tibet. *Res. Tibet. Archaeol. Cult. Reli.* **4**, 1–27 (2022).

42. D. B. Madsen, Conceptualizing the Tibetan Plateau: Environmental constraints on the peopling of the "third Pole". *Archaeol. Res. Asia.* **5**, 24–32 (2016).

43. J. Dunne, K. Rebay-Salisbury, R. B. Salisbury, A. Frisch, C. Walton-Doyle, R. P. Evershed, Milk of ruminants in ceramic baby bottles from prehistoric child graves. *Nature* **574**, 246–248 (2019).

44. C. Warinner, K. K. Richter, M. J. Collins, Paleoproteomics. *Chem. Rev.* **122**, 13401–13446 (2022).

45. Q. Qiu, G. J. Zhang, T. Ma, W. B. Qian, Z. Q. Ye, C. C. Cao, Q. J. Hu, J. Kim, D. M. Larkin, L. Auvil, B. Capitanu, J. Ma, H. A. Lewin, X. J. Qian, Y. S. Lang, R. Zhou, L. Z. Wang, K. Wang, J. Q. Xia, S. G. Liao, S. Pan, X. Lu, H. L. Hou, Y. Wang, X. T. Zang, Y. Yin, H. Ma, J. Zhang, Z. F. Wang, Y. M. Zhang, D. W. Zhang, T. Yonezawa, M. Hasegawa, Y. Zhong, W. B. Liu, Y. Zhang, Z. Y. Huang, S. X. Zhang, R. J. Long, H. M. Yang, J. A. Lenstra, D. N. Cooper, Y. Wu, J. Wang, P. Shi, J. Wang, J. Q. Liu, The yak genome and adaptation to life at high altitude. *Nat. Genet.* **44**, 946–949 (2012).

46. M. F. Kulyar, W. Y. Yao, Y. M. Ding, K. Li, L. H. Zhang, A. Y. Li, M. Waqas, H. C. Pan, M. Quan, Z. Zeng, K. Mehmood, S. L. Sizhu, J. K. Li, Bioactive potential of yak's milk and its products; pathophysiological and molecular role as an immune booster in antibiotic resistance. *Food Biosci.* **39**, 1–9 (2021).

47. L. F. Wang, Y. Ma, H. Li, F. M. Yang, J. J. Cheng, Identification and characterization of yak α-lactalbumin and β-lactoglobulin. *J. Dairy Sci.* **104**, 2520–2528 (2021).

48. J. D. Vigne, D. Helmer, Was milk a '"secondary product"' in the Old World Neolithisation process? Its role in the domestication of cattle, sheep and goats. *Anthropozoologica* **42**, 9–40 (2007).

49. F. H. Chen, F. Welker, C. C. Shen, S. E. Bailey, I. Bergmann, S. Davis, H. Xia, H. Wang, R. Fischer, S. E. Freidline, T. L. Yu, M. M. Skinner, S. Stelzer, G. R. Dong, Q. M. Fu, G. H. Dong, J. Wang, D. J. Zhang, J. J. Hublin, A late Middle Pleistocene Denisovan mandible from the Tibetan Plateau. *Nature* **569**, 409–412 (2019).

50. X. J. Zhang, K. E. Witt, M. M. Bañuelos, A. Ko, K. Yuan, S. H. Xu, R. Nielsen, E. Huerta-Sanchez, The history and evolution of the Denisovan-EPAS1 haplotype in Tibetans. *Proc. Natl. Acad. Sci. U.S.A.* **118**, 1–9 (2021).

51. D. J. Zhang, H. Xia, F. H. Chen, B. Li, V. Slon, T. Cheng, R. W. Yang, Z. Jacobs, Q. Y. Dai, D. Massilani, X. K. Shen, J. Wang, X. T. Feng, P. Cao, M. A. Yang, J. T. Yao, J. S. Yang, D. B. Madsen, Y. Y. Han, W. J. Ping, F. Liu, C. Perreault, X. S. Chen, M. Meyer, J. Kelso, S. Pääbo, Q. M. Fu, Denisovan DNA in Late Pleistocene sediments from Baishiya Karst Cave on the Tibetan Plateau. *Science* **370**, 584–587 (2020).

52. T. Cheng, D. J. Zhang, G. M. Smith, O. Jöris, J. Wang, S. L. Yang, H. Xia, X. K. Shen, Q. Li, X. S. Chen, D. P. Lin, Y. Y. Han, Y. S. Liu, M. R. Qiang, B. Li, F. H. Chen, Hominin occupation of the Tibetan Plateau during the Last Interglacial Complex. *Quat. Sci. Rev.* **265**, 107047 (2021).

53. D. D. Zhang, M. R. Bennett, H. Cheng, L. B. Wang, H. W. Zhang, S. C. Reynolds, S. D. Zhang, X. Q. Wang, T. Li, T. Urban, Q. Pei, Z. F. Wu, P. Zhang, C. R. Liu, Y. F. Wang, C. Wang, D. J. Zhang, R. Lawrence Edwards, Earliest parietal art: Hominin hand and foot traces from the middle Pleistocene of Tibet. *Sci. Bull.* **66**, 2506–2515 (2021).

54. X. L. Zhang, B. B. Ha, S. J. Wang, Z. J. Chen, J. Y. Ge, H. Long, W. He, W. Da, X. M. Nian, M. J. Yi, X. Y. Zhou, P. Q. Zhang, Y. S. Jin, O. Bar-Yosef, J. W. Olsen, X. Gao, The earliest human occupation of the high-altitude Tibetan Plateau 40 thousand to 30 thousand years ago. *Science* **362**, 1049–1051 (2018).

55. P. J. Brantingham, R. David, D. B. Madsen, Archaeology Augments Tibet’s genetic history. *Science* **329**, 1467 (2010).

56. L. L. Ren, G. H. Dong, F. W. Liu, J. d’Alpoim Guedes, R. K. Flad, M. M. Ma, H. M. Li, Y. S. Yang, Y. J. Liu, D. J. Zhang, G. L. Li, J. Y. Li, F. H. Chen, Foraging and farming: Archaeobotanical and zooarchaeological evidence for Neolithic exchange on the Tibetan Plateau. *Antiquity* **94**, 637–652 (2020).

57. Z. W. Zhang, Z. J. Chen, F. Marshall, H. L. Lu, X. Lemoine, T. Wangyal, T. Dorje, X. Y. Liu, The importance of localized hunting of diverse animals to early inhabitants of the Eastern Tibetan Plateau at the Neolithic site of Xiaoenda. *Quat. Int.* **529**, 38–46 (2019).

58. J. X. Song, Y. Y. Gao, L. Tang, Z. W. Zhang, M. H. Tang, H. L. Xu, T. Wangyal, H. B. Yuan, L. Li, Y. X. Li, S. Wangdue, X. Y. Liu, H. L. Lu, Farming and multi-resource subsistence in the third and second millennium BC: Archaeobotanical evidence from Karuo. *Archaeol. Anthropol. Sci.* **13**, 1–16 (2021).

59. F. H. Chen, J. F. Zhang, J. B. Liu, X. Y. Cao, J. Z. Hou, L. P. Zhu, X. K. Xu, X. J. Liu, M. D. Wang, D. Wu, L. X. Huang, T. Zeng, S. Zhang, W. Huang, X. Zhang, K. Yang, Climate change, vegetation history, and landscape responses on the Tibetan Plateau during the Holocene: A comprehensive review. *Quat. Sci. Rev.* **243**, 106444 (2020).

60. R. B. Harris, Rangeland degradation on the Qinghai-Tibetan plateau: A review of the evidence of its magnitude and causes. *J. Arid Environ.* **74**, 1–12 (2010).

61. C. S. Hughes, S. Moggridge, T. Müller, P. H. Sorensen, G. B. Morin, J. Krijgsveld, Single-pot, solid-phase-enhanced sample preparation for proteomics experiments. *Nat. Protoc.* **14**, 68–85 (2019).

62. E. Dunbar, G. T. Cook, P. Naysmith, B. G. Tripney, S. Xu, AMS ^{14}C dating at the scottish universities environmental research centre (SUERC) radiocarbon dating laboratory. *Radiocarbon* **58**, 9–23 (2016).

63. C. B. Ramsey, Methods for summarizing radiocarbon datasets. *Radiocarbon* **59**, 1809–1833 (2017).

64. P. J. Reimer, W. E. N. Austin, E. Bard, A. Bayliss, P. G. Blackwell, C. Bronk Ramsey, M. Butzin, H. Cheng, R. L. Edwards, M. Friedrich, P. M. Grootes, T. P. Guilderson, I. Hajdas, T. J. Heaton, A. G. Hogg, K. A. Hughen, B. Kromer, S. W. Manning, R. Muscheler, J. G. Palmer, C. Pearson, J. Van Der Plicht, R. W. Reimer, D. A. Richards, E. M. Scott, J. R. Southon, C. S. M. Turney, L. Wacker, F. Adolphi, U. Büntgen, M. Capano, S. M. Fahrni, A. Fogtmann-Schulz, R. Friedrich, P. Köhler, S. Kudsk, F. Miyake, J. Olsen, F. Reinig, M. Sakamoto, A. Sookdeo, S. Talamo, The IntCal20 Northern Hemisphere radiocarbon age calibration curve (0-55 cal kBP). *Radiocarbon* **62**, 725–757 (2020).

65. Y. L. Zhang, B. Y. Li, Z. Du, Datasets of the boundary and area of the Tibetan Plateau. *Acta Geogr. Sin.* **69**, 164–168 (2014).

66. E. Q. Xu, Land use of the Tibet Plateau in 2015 (version 1.0) (National Tibetan Plateau Data Center, 2019).

67. J. d'Alpoim Guedes, M. Aldenderfer, The archaeology of the early Tibetan plateau: New research on the initial peopling through the early bronze age. *J. Archaeol. Res.* **28**, 339–392 (2020).

68. D. Zheng, Q. S. Zhang, S. H. Wu, *Mountain Geoecology and Sustainable Development of the Tibetan Plateau* (Springer Science & Business Media, 2000).

69. A. Jarvis, H. I. Reuter, A. Nelson, E. Guevarra, Hole-filled seamless SRTM data V4 (International Centre for Tropical Agriculture, 2008), pp. 1–9; http://srtm.csi.cgiar.org.

70. E. Z. Tong, A summary of the archaeology of Tibet. *Wenwu* **9**, 9–19 (1985).

71. M. Aldenderfer, Y. N. Zhang, The prehistory of the tibetan plateau to the seventh century A.D.: Perspectives and Research From China and the West Since 1950. *J. World Prehistory* **18**, 1–55 (2004).

72. S. Wangdue, Z. Pu, Archaeological research in Tibet during the past 40 years. *Zhongguo Zangxue*, 201–212 (2005).

73. H. L. Lu, *Trans-Himalaya Interaction: An Archaeological Study of the Prehistoy of Western Tibet* (Science Press, 2015).

74. Y. X. Li, The survey report of Gebusailu Burial Site in Zanda County, Tibet. *Kaogu*, 39–44 (2001).

75. Sichuan University, Cultural Heritage Bureau of Tibet Autonomous Region, in *The Excavation Report of the Piyang Dongga Site* (Sichuan People's Publishing House, 2008).

76. H. Wang, Y. X. Li, Y. M. Feng, L. L. Tu, H. L. Zhang, The age of human bones from Redilong, Qamdo County Tibet. *Acta Geosci. Tica Sin.* **24**, 569–572 (2003).

77. Y. H. He, L. H. Cai, W. Du, X. H. Yu, C. Y. Ma, P. Li, Report on the excavation of the cist burials at the Pukar Gongma Site, ‘Bri stod County, Qinghai Province. *Zangxue Xuekan* **1**, 24–41 (2013).

78. L. S. Weyrich, S. Duchene, J. Soubrier, L. Arriola, B. Llamas, J. Breen, A. G. Morris, K. W. Alt, D. Caramelli, V. Dresely, M. Farrell, A. G. Farrer, M. Francken, N. Gully, W. Haak, K. Hardy, K. Harvati, P. Held, E. C. Holmes, J. Kaidonis, C. Lalueza-Fox, M. De La Rasilla, A. Rosas, P. Semal, A. Soltysiak, G. Townsend, D. Usai, J. Wahl, D. H. Huson, K. Dobney, A. Cooper, Neanderthal behaviour, diet, and disease inferred from ancient DNA in dental calculus. *Nature* **544**, 357–361 (2017).

79. A. Gismondi, A. D’Agostino, G. Di Marco, C. Martínez-Labarga, V. Leonini, O. Rickards, A. Canini, Back to the roots: Dental calculus analysis of the first documented case of coeliac disease. *Archaeol. Anthropol. Sci.* **12**, 6 (2020).

80. K. Hardy, A. Radini, S. Buckley, R. Blasco, L. Copeland, F. Burjachs, J. Girbal, R. Yll, E. Carbonell, J. M. Bermúdez de Castro, Diet and environment 1.2 million years ago revealed through analysis of dental calculus from Europe’s oldest hominin at Sima del Elefante, Spain. *Sci. Nat.* **104**, 2 (2017).

81. C. Warinner, J. F. M. Rodrigues, R. Vyas, C. Trachsel, N. Shved, J. Grossmann, A. Radini, Y. Hancock, R. Y. Tito, S. Fiddyment, C. Speller, J. Hendy, S. Charlton, H. U. Luder, D. C. Salazar-García, E. Eppler, R. Seiler, L. H. Hansen, J. A. S. Castruita, S. Barkow-Oesterreicher, K. Y. Teoh, C. D. Kelstrup, J. V. Olsen, P. Nanni, T. Kawai, E. Willerslev, C. Von Mering, C. M. Lewis Jr., M. J. Collins, M. T. P. Gilbert, F. Rühli, E. Cappellini, Pathogens and host immunity in the ancient human oral cavity. *Nat. Genet.* **46**, 336–344 (2014).

82. C. Warinner, C. Speller, M. J. Collins, A new era in palaeomicrobiology: Prospects for ancient dental calculus as a long-term record of the human oral microbiome. *Philos. Trans. R. Soc. B Biol. Sci.* **370**, 20130376 (2015).

83. C. J. Adler, K. Dobney, L. S. Weyrich, J. Kaidonis, A. W. Walker, W. Haak, C. J. A. Bradshaw, G. Townsend, A. Sołtysiak, K. W. Alt, J. Parkhill, A. Cooper, Sequencing ancient calcified dental plaque shows changes in oral microbiota with dietary shifts of the Neolithic and Industrial revolutions. *Nat. Genet.* **45**, 450–455 (2013).

84. L. Granehäll, K. D. Huang, A. Tett, P. Manghi, A. Paladin, N. O'Sullivan, O. Rota-Stabelli, N. Segata, A. Zink, F. Maixner, Metagenomic analysis of ancient dental calculus reveals unexplored diversity of oral archaeal Methanobrevibacter. *Microbiome* **9**, 1–18 (2021).

85. A. R. Lieverse, Diet and the aetiology of dental calculus. *Int. J. Osteoarchaeol.* **9**, 219–232 (1999).

86. A. Radini, E. Nikita, S. Buckley, L. Copeland, K. Hardy, Beyond food: The multiple pathways for inclusion of materials into ancient dental calculus. *Am. J. Phys. Anthropol.* **162**, 71–83 (2017).

87. A. Scott, R. C. Power, V. Altmann-Wendling, M. Artzy, M. A. S. Martin, S. Eisenmann, R. Hagan, D. C. Salazar-García, Y. Salmon, D. Yegorov, I. Milevski, I. Finkelstein, P. W. Stockhammer, C. Warinner, Exotic foods reveal contact between South Asia and the Near East during the second millennium BCE. *Proc. Natl. Acad. Sci. U.S.A.*, **118**, e2014956117 (2021).

88. R. C. Power, D. C. Salazar-García, R. M. Wittig, M. Freiberg, A. G. Henry, Dental calculus evidence of Taï Forest Chimpanzee plant consumption and life history transitions. *Sci. Rep.* **5**, 15161 (2015).

89. A. Akcalı, N. P. Lang, Dental calculus: The calcified biofilm and its role in disease development. *Periodontol. 2000* **76**, 109–115 (2018).

90. I. M. Velsko, K. A. Overmyer, C. Speller, L. Klaus, M. J. Collins, L. Loe, L. A. F. Frantz, K. Sankaranarayanan, C. M. Lewis, J. B. R. Martinez, E. Chaves, J. J. Coon, G. Larson, C. Warinner, The dental calculus metabolome in modern and historic samples. *Metabolomics* **13**, 1–17 (2017).

91. M. Mackie, J. Hendy, A. D. Lowe, A. Sperduti, M. Holst, M. J. Collins, C. F. Speller, Preservation of the metaproteome: Variability of protein preservation in ancient dental calculus. *Sci. Technol. Archaeol. Res.* **3**, 58–70 (2017).

92. J. Hendy, F. Welker, B. Demarchi, C. Speller, C. Warinner, M. J. Collins, A guide to ancient protein studies. *Nat. Ecol. Evol.* **2**, 791–799 (2018).

93. J. Hendy, Ancient protein analysis in archaeology. *Sci. Adv.* **7**, 1–12 (2021).

94. Z. W. Zhang, S. Wangdue, H. L. Lu, S. C. Nyima, Identification and Interpretation of Faunal Remains from a Prehistoric Cist Burial in Amdo County North Tibet. *J. Tibetol.* **12**, 1–18 (2015).

95. T. Tong, L. H. Li, S. Huang, The excavation of Gurugyam cemetery in Gar County, Ngari Prefercture, Tibet Atonomous Region in 2012. *Kaogu Xuebao*, 563–587 (2014).

96. J. X. Song, H. L. Lu, Z. Z. Zhang, X. Y. Liu, Archaeobotanical remains from the mid-first millennium AD site of Kaerdong in western Tibet. *Archaeol. Anthropol. Sci.* **10**, 2015–2026 (2018).

97. M. Aldenderfer, J. T. Eng, *Death and Burial Among Two Ancient High Altitude Communities of Nepal* (John Wiley and Sons, 2016).

98. L. L. Ren, G. H. Dong, H. M. Li, D. Rhode, R. K. Flad, G. Q. Li, Y. Yang, Z. X. Wang, L. H. Cai, X. Y. Ren, D. J. Zhang, F. H. Chen, Dating human settlement in the east-central tibetan plateau during the late holocene. *Radiocarbon* **60**, 137–150 (2018).

99. D. P. Agrawal, J. Kharakwal, S. Kusumgar, M. G. Yadava, Cist burials of the Kumaun Himalayas. *Antiquity* **69**, 550–554 (1995).

100. H. L. Lu, X. Z. Chen, Z. W. Zhang, L. Tang, X. Lemoine, S. Wangdue, Z. J. Chen, X. Y. Liu, M. D. Frachetti, Early agropastoral settlement and cultural change in central Tibet in the first millennium BC: Excavations at Bangga. *Antiquity*, 1–18 (2021).

101. T. Tong, L. H. Li, C. Cilie, J. Yao, Excavations at Chuvtag and Gurugyam cemeteries. *Kaogu* **7**, 29–50 (2015).

102. D. Chen, Z. Ciren, K. Zhang, The ancient tombs at Jinlinlong in Ganzi County Sichuan. *Kaogu* **1**, 28–36 (1986).

103. B. X. Zhou, Faunal remains from Qugong Site, in *Qugong in Lhasa* (Encyclopedia of China Publishing House, 1999).

104. W. Chen, Analysis of the Kashahu Burial Site. *Sichuan wenwu*, 36–42 (2011).

105. G. H. Dong, L. L. Ren, X. Jia, X. Y. Liu, S. M. Dong, H. M. Li, Z. X. Wang, Y. M. Xiao, F. H. Chen, Chronology and subsistence strategy of Nuomuhong Culture in the Tibetan Plateau. *Quat. Int.* **426**, 42–49 (2016).

106. Z. W. Zhang, *Analysis of Animal Bones Unearthed from Ashaonao Site* (Bashu Publishing House, 2017).

107. K. Y. He, J. Chen, X. Z. Chen, Y. G. Fan, J. Li, Analysis of animal bones recovered from Shidaqiu site in Maerkang. *Chengdu kaogu faxian*, 295–303 (2012).

108. H. Liu, L. Tang, The laolongtou cemetery and the Yanyuan bronzes. *J. Natl. Museum China* **6**, 22–29 (2006).

109. L. Li, "Research on the utilization of animal resources at Changning, Qinghai Province." thesis, Jilin University (2012).

110. L. L. Ren, "A study on animal exploitation strategies from the late Neolithic to Bronze Age in northeastern Tibetan Plateau and its surrounding areas, China," thesis, Lanzhou University (2017).

111. J. Yuan, M. F. Yang, *The Siwa Culture Cemetery of Xujianian* (Science Press, 2006), pp. 238–244.

112. K. Brunson, L. L. Ren, Z. Xin, D. Xiaoling, W. Hui, Z. Jing, R. Flad, Zooarchaeology, ancient mtDNA, and radiocarbon dating provide new evidence for the emergence of domestic cattle and caprines in the Tao River Valley of Gansu Province, northwest China. *J. Archaeol. Sci. Rep.* **31**, 102262 (2020).

113. Y. R. Wang, "Identifying the beginnings of sheep husbandry in western China," thesis, University of Cambridge (2017).

114. L. W. Fu, *Ganguya in Jiuquan* (Cultural Relics Press, 2016).

115. G. Q. Qi, *Archaeology at Donghuishan, Minle–Revelations and Research at the Siba Culture Cemetery* (Science Press, 1998).

116. P. Lu, J. Yuan, Exchange and transformation–A preliminary study of Pre-Qin agricultural production modes in the upper reaches of the Yellow River (part 1). *Nanfang Wenwu*, 170–179 (2018).

117. Y. B. Song, G. K. Chen, H. Wang, X. J. Fan, G. Y. Jin, Analysis of animal remains unearthed from Xichengyi, Zhangye in 2014. *Dongfang Kaogu*, 233–242 (2016).
